# How NeuroArts Aid in the Well-Being of People: A Scoping Review

**DOI:** 10.7759/cureus.109729

**Published:** 2026-05-27

**Authors:** Jon Stewart H Dy, Justine Grace E Regala, Kasandra Camelle B Tan, Bernard F Laya, Joven R Cuanang

**Affiliations:** 1 Neurosciences, El Refugio Arts and Sciences Foundation, Inc., Antipolo, PHL; 2 Neurosciences, Mapua University School of Medicine, Makati City, PHL; 3 Neurology, Chinese General Hospital and Medical Center, Manila City, PHL; 4 Neurosciences, St. Luke's Medical Center College of Medicine - William H. Quasha Memorial, Quezon City, PHL; 5 Neurosciences, Philippine Institute for NeuroArts, Antipolo, PHL; 6 Undergraduate Medical Education, St. Luke's Medical Center College of Medicine - William H. Quasha Memorial, Quezon City, PHL; 7 Undergraduate Medical Education, University of the East Ramon Magsaysay Memorial Medical Center College of Medicine, Quezon City, PHL; 8 Radiology, St. Luke's Medical Center-Global City, Taguig, PHL; 9 Radiology, St. Luke's Medical Center-Quezon City, Quezon City, PHL; 10 Institute for Neurosciences, St. Luke's Medical Center, Quezon City, PHL

**Keywords:** arts in health, mental health, neuroaesthetics, neuroarts, scoping review, well-being

## Abstract

NeuroArts is an emerging interdisciplinary field examining how creative and aesthetic experiences influence neural, physiological, and behavioral processes related to health and well-being. This study aimed to map and synthesize existing evidence on arts-based interventions and their impact on well-being across healthy and clinical populations. A scoping review was conducted from May to November 2025 in accordance with PRISMA-ScR guidelines. Studies published between January 2000 and November 2025 involving arts-based interventions in healthy or clinical populations were included. Literature searches were performed using PubMed, Scopus, Cochrane Library, ClinicalTrials.gov, and HERDIN databases. Titles, abstracts, and full texts were screened independently according to predefined eligibility criteria, and relevant study characteristics and outcomes were extracted. Included studies were categorized based on the World Health Organization classifications of art forms. A total of 9,614 records were identified, with 67 studies meeting the inclusion criteria. Arts-based interventions - including music, dance, visual arts, literature, and cultural engagement - were associated with improvements in psychological well-being, cognitive function, and quality of life across populations. NeuroArts provides a useful framework for understanding how arts-based practices contribute to health and well-being in both clinical and community contexts. Findings support the integration of arts-based interventions into therapeutic and health-promoting settings, with implications for clinical practice, policy, and future research.

## Introduction and background

The arts have long been recognized as contributing to human health and well-being, with effects spanning physical, psychological, and social domains [1]. In recent years, there has been increasing interest in examining these benefits through a neuroscientific perspective, giving rise to the field of NeuroArts [2,3]. NeuroArts is an emerging interdisciplinary field that examines how experiences derived from creativity, the arts, and aesthetics influence neural activity, physiological responses, cognition, emotion, and behavior, and how these effects may contribute to health and well-being [2,3].

Drawing from neuroscience, psychology, empirical aesthetics, rehabilitation medicine, and the arts, NeuroArts explores the biological and behavioral mechanisms through which artistic experiences affect the human brain and body. These experiences may include engagement with music, dance, visual arts, literature, cultural activities, and digital or electronic arts, among others. Emerging evidence suggests that such interventions may influence emotional regulation, stress responses, neuroplasticity, cognition, social connectedness, and quality of life across both healthy and clinical populations [1-3].

Despite this growing interest, NeuroArts remains an evolving field with ongoing efforts to clarify its conceptual foundations. Early descriptions characterize NeuroArts as the study of how artistic and aesthetic experiences influence brain function and contribute to measurable changes in health and well-being [1]. These effects are thought to involve complex interactions across emotional, cognitive, and social processes [2,3].

The World Health Organization (WHO) has categorized artistic engagement into multiple domains, including performing arts (e.g., music, singing, and dance), visual arts and crafts (e.g., painting, pottery, embroidery, and clay art), literature (e.g., reading, writing, storytelling, and poetry), culture (e.g., museum and gallery engagement), and digital or electronic arts (e.g., online art exposure, digital storytelling, and video-based creative expression) [3]. This framework provides a useful structure for examining the diverse mechanisms through which artistic activities may affect health outcomes across different populations [3].

While individual studies and reviews have explored the health-related effects of specific art forms, there remains a need for a comprehensive synthesis that integrates findings across modalities. In particular, there is limited consolidation of evidence examining NeuroArts as a unified concept, especially in relation to both healthy individuals and those with clinical conditions. Given the broad, interdisciplinary, and heterogeneous nature of the existing literature, a scoping review methodology was considered appropriate to map the available evidence, identify major NeuroArts domains, and synthesize reported health-related outcomes across diverse study designs and populations.

This review sought to address the following research questions: (1) What forms of NeuroArts interventions have been studied in healthy and clinical populations? (2) What health and well-being outcomes have been associated with these interventions? (3) How has NeuroArts been conceptually defined and categorized in the existing literature?

Accordingly, this scoping review aimed to map and synthesize existing evidence on arts-based interventions and their impact on well-being across healthy and clinical populations.

## Review

Methods

A scoping review was performed from May to November 2025 in order to identify and map the available evidence on NeuroArts. A formal review protocol was not prospectively registered. Studies examining interventions involving various art forms, as defined by the World Health Organization, across both outpatient and inpatient settings were identified. In order to follow a standardized process, the Preferred Reporting Items for Systematic Reviews and Meta-Analyses extension for Scoping Reviews (PRISMA-ScR) guidelines was utilized.

Data Sources

International and local medical databases were used in searching relevant literature, including PubMed, SCOPUS, ClinicalTrials.gov, Cochrane and Health Research and Development Information Network (HERDIN). The search terms (“painting” OR “sculpture” OR “drawing” OR “textile” OR “crafts” OR “singing” OR “dancing” OR “musical instrument” OR “drama” OR “performing in a play” OR “reading for pleasure” OR “creative writing” OR “composing music” OR “stories” OR “story-telling”) AND (“effect on health” OR “mental health” OR “quality of life” OR “disease” OR “disorder”) were used and combined. A detailed electronic search strategy for at least one database is provided in the appendix/supplementary materials. Titles and abstracts were then evaluated and screened based on the eligibility criteria. Duplicates were excluded. Full-text articles were also requested and reviewed.

Study Selection

Eligibility criteria for the search included English-language publications in peer-reviewed journals from January 2000 through November 2025.

Inclusion criteria included: (1) systematic reviews, meta-analyses, randomized controlled trials (RCTs), retrospective cohort studies, case series, and case reports; (2) studies involving various art forms as defined by the World Health Organization; (3) studies involving human participants in both healthy and clinical populations; (4) studies evaluating health-related outcomes, including psychological, cognitive, behavioral, physiological, or quality-of-life outcomes; and (5) studies discussing the definitions of NeuroArts and its applications relevant to health and well-being.

Exclusion criteria included: (1) studies involving animal populations; and (2) non-English-language publications.

The search for specific art forms or study designs was not restricted in order to capture data on all available art forms as defined by the World Health Organization.

Data Extraction

Data extraction was conducted in accordance with the Preferred Reporting Items for Systematic Reviews and Meta-Analyses extension for Scoping Reviews (PRISMA-ScR) framework. Pertinent information regarding the various art forms and their effects on health and well-being was obtained from the included studies. Extracted variables included authors, publication year, study design, study population, NeuroArts modality, intervention characteristics, study setting, and reported health-related outcomes. Authors, titles, and institutional affiliations were likewise obtained from the published studies.

The data were analyzed and evaluated using a qualitative content approach involving the systematic organization and synthesis of findings to identify key concepts and patterns across studies. Results were summarized and presented in figures and tables where appropriate. To minimize potential bias, two reviewers were involved in the process, and discrepancies among the included studies were regularly discussed and resolved through consensus. Detailed study characteristics and extracted data are summarized in the results tables and supplementary materials where appropriate.

Results

Search of Studies and Study Characteristics

A comprehensive literature search was conducted using PubMed, SCOPUS, ClinicalTrials.gov, Cochrane Library, and the Health Research and Development Information Network (HERDIN). A representative PubMed search strategy is provided in the Supplementary Material (see Appendices).

Database searching yielded a total of 9,614 records. Specifically, records identified included PubMed (n = 9,319), SCOPUS (n = 0), ClinicalTrials.gov (n = 8), Cochrane Library (n = 253), and HERDIN (n = 34). No additional records were identified through other sources.

Prior to screening, 80 duplicate records were removed. Automated screening tools were additionally utilized to exclude 2,057 records that were considered ineligible based on predefined criteria, including non-health-related content, non-human studies, and records unrelated to NeuroArts domains. The remaining 7,477 records underwent title and abstract screening according to the eligibility criteria. Of these, 6,628 records were excluded.

**Figure 1:**
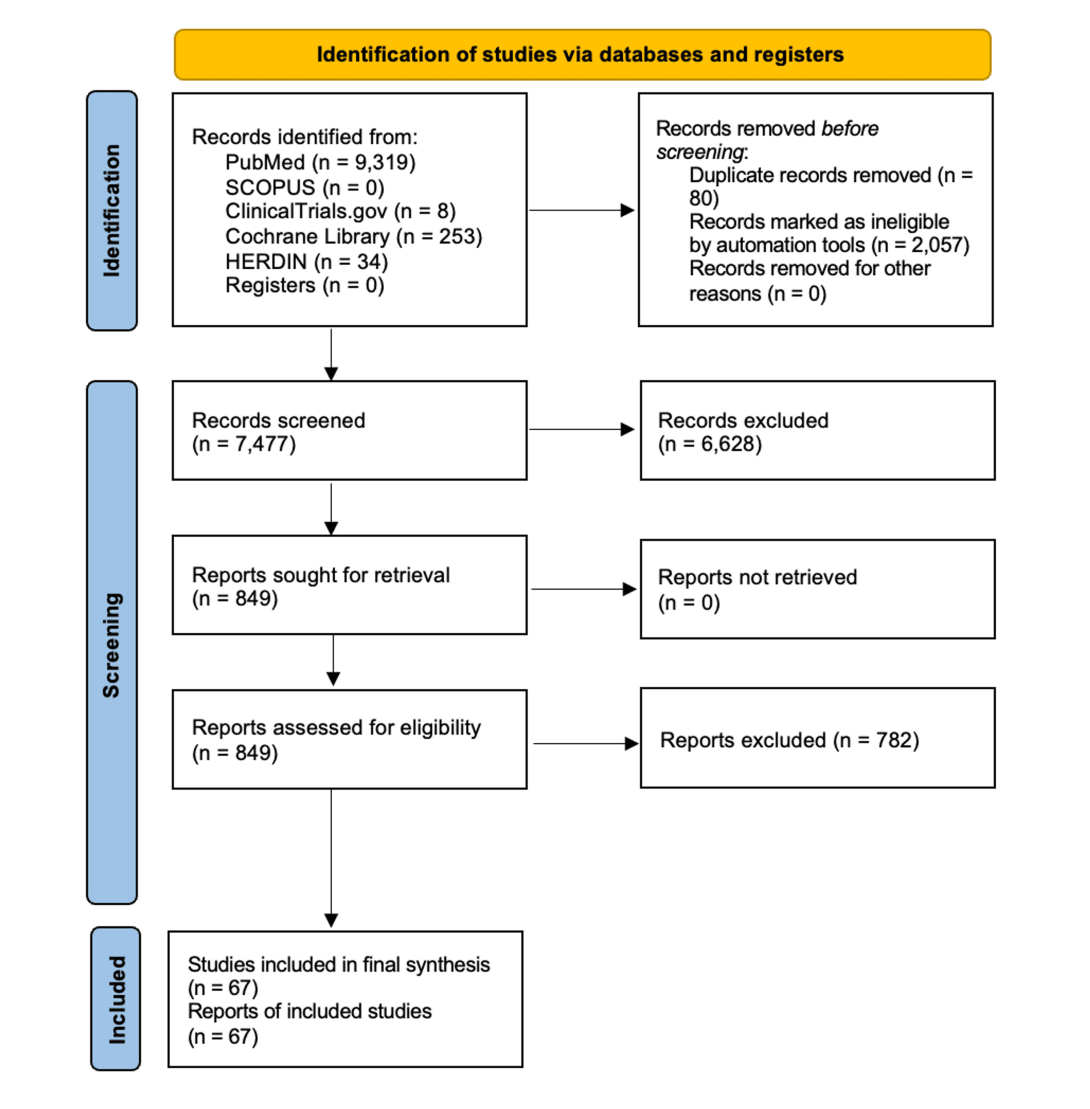
PRISMA-ScR flow diagram illustrating the study selection process from PubMed, Scopus, Cochrane, ClinicalTrials.gov, and HERDIN. PRISMA-ScR = Preferred Reporting Items for Systematic reviews and Meta-Analyses extension for Scoping Reviews

A total of 849 full-text reports were sought for retrieval and assessed for eligibility. Of these, 782 reports were excluded for reasons including lack of relevance to NeuroArts domains, absence of health-related outcomes, non-original article types, non-human studies, or failure to meet predefined inclusion criteria. Ultimately, 67 studies met the eligibility criteria and were included in the final synthesis (Figure 1). Included studies were subsequently categorized according to NeuroArts domains and population type (healthy versus clinical populations). Condensed study characteristics are summarized in the main results tables, while expanded reviewer-responsive extraction tables detailing study populations, intervention characteristics, settings, and outcomes are provided in the Supplementary Material (see Appendices).

**Figure 2:**
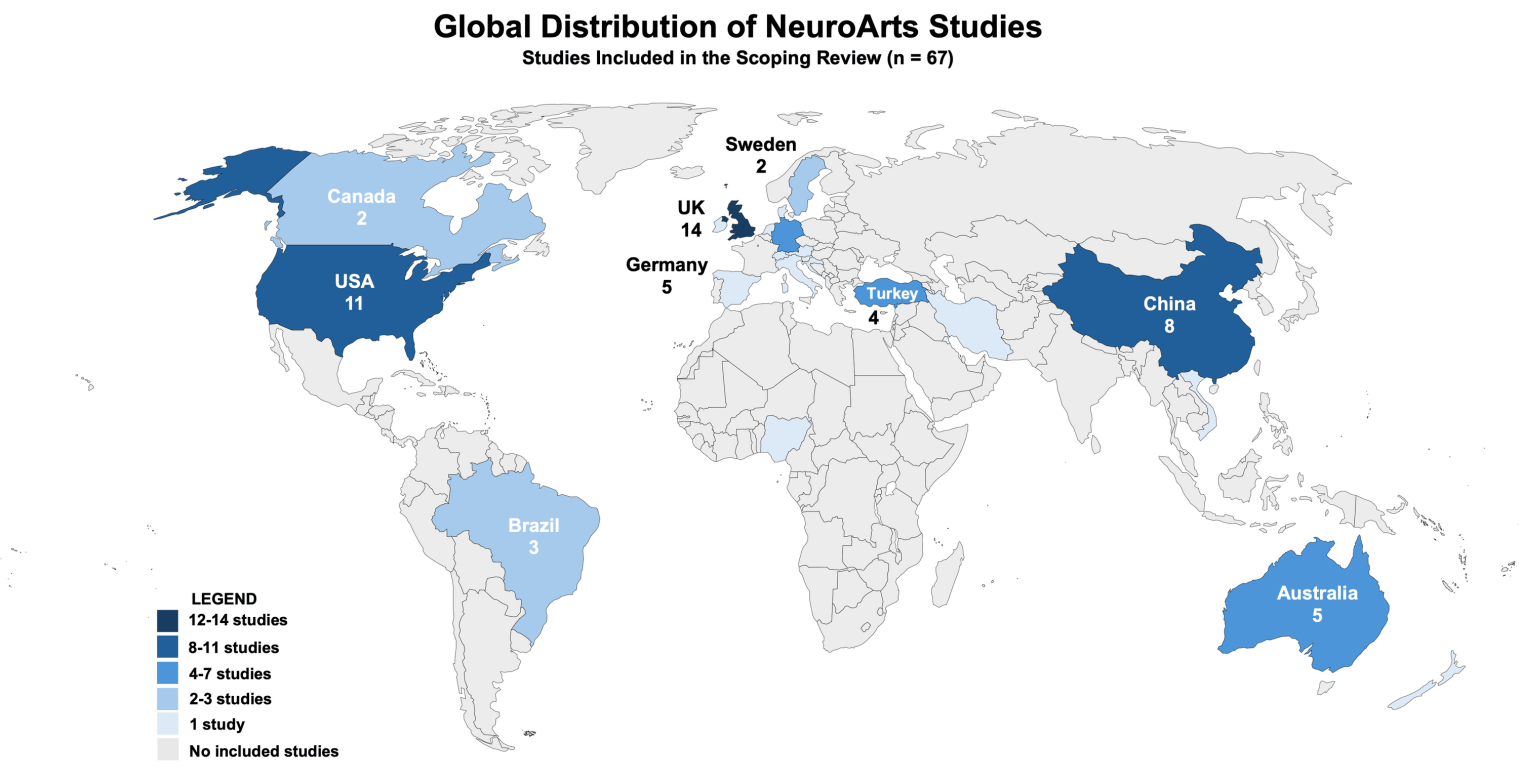
Global distribution of studies included in the NeuroArts scoping review (n = 67). Studies were conducted across 24 countries, with the highest concentration in the United Kingdom (n = 14), United States (n = 11), and China (n = 8). Countries are shaded according to the number of studies contributed. Image credit: Created by the authors using Microsoft PowerPoint (Microsoft Corporation, Redmond, Washington, USA)

The 67 included studies were conducted across 24 countries (Figure 2). The United Kingdom contributed the largest number of studies (n = 14), followed by the United States (n = 11) and China (n = 8). Germany and Australia each contributed five studies, while Turkey contributed four studies. Brazil contributed three studies, and Sweden and Canada contributed two studies each. The remaining countries each contributed one study.

Art Forms and Approaches

The included studies encompassed a broad range of artistic modalities. Performing arts was the most frequently investigated category (n = 32), followed by visual arts, design and craft (n = 13) and literature-based interventions (n = 12). Cultural engagement (n = 7) and online, digital, and electronic arts (n = 3) were less commonly studied.

Across all modalities, randomized controlled trials (RCTs) represented the largest proportion of study designs (n = 24), particularly within performing arts interventions. The characteristics of included studies are summarized in Table 1 and detailed further in the Supplementary Material.

Outcomes in Healthy and Clinical Populations

Performing arts (n = 32): Among healthy populations, performing arts interventions demonstrated benefits across both physiological and psychosocial domains. Music and singing-based activities were linked to increased pain tolerance, improved emotional well-being, reduced stress levels, and enhanced social connectedness [4-6]. Dance interventions were associated with gains in global cognition, memory, and overall physical health [7-10]. Additionally, group-based performing arts activities contributed to improvements in social bonding, subjective well-being, and creativity (Table 1) [9].

**Table 1:** Primary studies evaluating efficacy of Performing Arts in Healthy Populations

Author (Year)	Country	Study Design	Study Population	NeuroArts Modality	Setting	Outcomes
Likamwa et al. (2022) [4]	United States	Quasi-experimental	Healthy adults	Music and singing (active vocalization)	Laboratory setting	Singing delayed pain onset, increased pain tolerance, and was associated with higher self-reported singing proficiency compared to passive listening or silence
Wulff et al. (2021) [5]	Germany	Randomized Controlled Trial	Pregnant women	Music (receptive)	Clinical/Antenatal setting	Music listening improved emotional state, enhanced maternal–fetal bonding, and reduced stress levels
de Witte et al. (2022) [6]	Netherlands	Systematic review & Meta-analysis	Mixed populations (clinical and non-clinical)	Music therapy	Clinical and community settings	Music therapy demonstrated a medium-to-large effect in reducing stress-related outcomes, with stronger effects in controlled settings and non-Western populations
Tunçgenç et al. (2024) [7]	United Kingdom	Randomized Controlled Trial	Adolescents	Dance/movement (synchrony-based)	Online/virtual setting	Online group dance intervention enhanced social bonding, prosocial behavior, well-being, and positive future outlook among adolescents
Wind et al. (2020) [8]	Brazil	Randomized Controlled Trial	Dance-inexperienced female	Dance/movement	Controlled experimental setting	Dance intervention increased brain activity and enhanced functional connectivity associated with motor and cognitive processes
Agres & Chen (2025) [9]	Singapore	Randomized Controlled Trial	University students	Performing arts (facilitated drumming and dancing)	University / community-based setting	The 10-week Movin’ and Groovin’ for Wellness programme improved mental health, quality of life, and creativity, with qualitative benefits in social connection and creative expression.
Hwang & Braun (2015) [10]	United States	Systematic review	Older adults (≥60 years)	Dance/movement	Multiple settings (community and clinical)	Dance interventions improved balance, strength, flexibility, and cognitive function, with consistent positive effects on functional fitness in older adults

In clinical populations, music-based interventions were the most extensively studied and showed consistent benefits across a range of conditions. In post-surgical settings, music exposure was associated with improved recovery trajectories, reduced recovery time, and enhanced overall well-being [11]. Among patients undergoing chemotherapy, music interventions were linked to reductions in fatigue, depression, and anxiety, alongside improvements in quality of life [12,13]. Singing interventions in respiratory disease populations were associated with decreased depressive symptoms and improved respiratory function [14,15]. Additional evidence also supports improvements in exercise capacity following structured music-based respiratory interventions [16]. In neurodegenerative conditions such as Alzheimer’s disease, music therapy was associated with improvements in cognition, behavior, and functional status [17,18]. Additional neurological benefits were observed in stroke and other neurological conditions, where music-based interventions were linked to improvements in language recovery, gait, overall neurological function, and physical health outcomes in chronic disease populations [19-21]. Group singing interventions have also demonstrated improvements in symptoms, well-being, and quality of life among patients with cancer and their caregivers [22]. Dance-based interventions likewise demonstrated benefits across multiple clinical groups. In individuals with cerebral palsy, structured dance programs improved the range of motion [23]. Among individuals with profound intellectual and multiple disabilities (PIMD), water-based dance interventions were associated with reductions in stress and muscular hypertonia [24]. In Parkinson’s disease, dance interventions were linked to improvements in motor function, quality of life, and depressive symptoms [25-28]. However, outcomes were not universally positive, as some studies reported no significant changes in immune function, quality of life, or sleep in specific populations (Table 2) [29-31].

**Table 2:** Primary studies evaluating efficacy of Performing Arts in Clinical Populations

Author (Year)	Country	Study Design	Study Population	NeuroArts Modality	Setting	Outcomes
Vetter et al. (2015) [11]	Switzerland	Systematic review & Meta-analysis	Surgical adult patients	Music and visual/ambient arts	Clinical (perioperative) setting	Art interventions, particularly music, significantly reduced pain, anxiety, blood pressure, and heart rate; self-selected music showed stronger effects
Lima et al. (2020) [12]	Brazil	Randomized Controlled Trial	Breast cancer patients undergoing chemotherapy	Music (receptive intervention)	Clinical (oncology/chemotherapy setting)	Music intervention improved quality of life and reduced anxiety, depression, fatigue, and chemotherapy-related symptoms (e.g., vomiting)
Nguyen et al. (2023) [13]	Vietnam	Randomized Controlled Trial	Women with breast and gynecologic cancer undergoing chemotherapy	Music (receptive) + relaxation (combined intervention)	Clinical (oncology setting)	Combined music and progressive muscle relaxation reduced anxiety, depression, and stress, and improved quality of life compared to control.
Philip et al. (2020) [14]	United Kingdom	Randomized Controlled Trial	Patients with Chronic Obstructive Pulmonary Disease	Music (active; group singing)	Clinical + community (online and face-to-face)	Group singing improved depression and balance confidence, with additional psychosocial benefits and feasibility for online delivery
Lewis et al. (2016) [15]	United Kingdom	Systematic review	Patients with Chronic Obstructive Pulmonary Disease	Music (active; group singing)	Clinical + community	Singing interventions improved health-related quality of life and anxiety, with consistent patient-reported psychosocial benefits
Okur and Nural (2025) [16]	Turkey	Randomized Controlled Trial	Patients with Chronic Obstructive Pulmonary Disease	Music (active; singing and instrument-playing [melodica])	Clinical (hospital setting)	Singing or playing melodica reduced symptoms (dyspnea, fatigue, anxiety) and improved self-efficacy and exercise capacity
Gomez-Gallego et al. (2021) [17]	Spain	Randomized controlled trial	Patients with Alzheimer’s disease	Music (active vs receptive)	Clinical (nursing home setting)	Active music intervention significantly improved cognition, behavior, and functional status more than receptive music and usual care
Bleibel et al. (2023) [18]	Lebanon	Systematic review	Patients with Alzheimer’s disease	Music therapy	Clinical setting	Music therapy improved cognitive functions (memory, attention, language, and global cognition), with stronger effects observed in active music participation
Liu et al. (2022) [19]	China	Systematic review & Meta-analysis	Patients with post-stroke Aphasia	Music (active; melodic intonation therapy/singing-based)	Clinical (neurorehabilitation setting)	Music therapy improved functional communication, repetition, and naming, but not comprehension in post-stroke aphasia
Wang et al. (2021) [20]	China	Prospective controlled clinical study	Patients with ischemic stroke	Music (rhythmic auditory stimulation)	Clinical (stroke rehabilitation setting)	Music rhythm–based therapy improved gait parameters, balance, motor function, and rehabilitation outcomes compared to control
Qin (2020) [21]	China	Randomized Controlled Trial	Patients with ankylosing spondylitis	Music (receptive; traditional Chinese) ± comparison with painting	Clinical (hospitalized setting)	Music therapy improved physical and psychological outcomes (including emotional state, cognition, and self-esteem) more than painting therapy and routine care
Bains et al. (2024) [22]	Australia	Systematic review	Patients with cancer and their caregivers	Music (active; group singing)	Clinical/community	Group singing reduced anxiety, improved well-being, self-efficacy, and quality of life, with benefits observed in patients and caregivers
Sihvonen et al. (2017) [23]	Finland	Narrative review	Patients with neurological disorders (e.g., stroke, dementia, Parkinson’s disease)	Music (receptive and active; listening, singing, instrument playing)	Clinical (neurorehabilitation)	Music-based interventions improve cognition, motor function, and emotional well-being, with shared neural mechanisms involving reward, arousal, learning, and neuroplasticity
Teixeira-Machado & DeSantana (2019) [24]	Brazil	Randomized Controlled Trial	Young people with cerebral palsy	Dance (active movement-based)	Clinical (rehabilitation setting)	Dance intervention improved lower-limb range of motion and overall motor function compared to control
Lundqvist et al. (2022) [25]	Sweden	Randomized crossover trial	Adults with profound intellectual and multiple disabilities	Dance (water-based; structured movement)	Clinical/community (rehabilitation setting)	Water-based dance intervention improved well-being, social interaction, and physiological outcomes (including stress reduction)
Carapellotti et al. (2020) [26]	United Kingdom	Systematic review & Meta-analysis	Adults diagnosed with Parkinson's disease	Dance (active movement-based)	Clinical/community (rehabilitation setting)	Dance interventions improved motor symptoms (especially balance and motor severity), with emerging benefits on non-motor symptoms and quality of life
Walton et al. (2022) [27]	Sweden	Mixed-methods feasibility study	Patients with Parkinson's Disease	Dance (active; digital/online delivery)	Community (online/remote setting)	Digital dance intervention was feasible and associated with improvements in quality of life, psychological well-being, and cognitive complaints
Tao et al. (2023) [28]	China	Systematic scoping review and meta-analysis	Older adults with mild cognitive impairment, Alzheimer’s disease, and dementia	Dance (active; dance movement interventions)	Clinical/community	Dance interventions improved global cognition, memory, balance, and depression, with no significant effect on executive function
Barnish & Barran (2020) [29]	United Kingdom	Systematic review	Patients with Parkinson’s disease	Multimodal (dance, singing, music therapy, theatre)	Clinical/community	Performing arts interventions improved quality of life, motor function, communication, and cognitive outcomes across multiple modalities
Salihu et al. (2021) [30]	Nigeria	Quasi-experimental	Internally Displaced Persons (IDP) with depressive symptoms	Dance (active; African circle dance)	Community (IDP camp setting)	Dance intervention significantly reduced depressive symptoms and stress compared to control.
Pisu et al. (2017) [31]	United States	Randomized Controlled Trial	Women who were at least three months post-completion of primary cancer treatment	Dance (active; ballroom/partner-based)	Clinical/community	Dance intervention improved quality of life, physical activity, vitality, and dyadic relationship outcomes (e.g., trust)
Ghayomzadeh et al. (2019) [32]	Iran	Quasi-experimental	Women living with HIV (WLWH)	Dance (active; aerobic dance)	Clinical/community	Aerobic dance improved psychological well-being and immune function indicators in women with HIV
Davico et al. (2022) [33]	Italy	Scoping review	At-risk populations and gatekeepers	Performing arts (music, dance, drama, role-playing)	Community and public health settings	Performing arts interventions improved awareness, self-efficacy, and soft skills relevant to suicide prevention; evidence supports feasibility but limited causal inference
McCrary et al. (2022) [34]	Germany	Systematic review & Meta-analysis	Mixed populations (n = 779 across 26 studies)	Music interventions (listening, therapy, singing)	Clinical and community settings	Music interventions were associated with significant improvements in mental and physical components of health-related quality of life (HRQOL), with clinically meaningful effect sizes
Hauck et al. (2013) [35]	Germany	Quasi-experimental	Healthy female participants	Music (receptive and active/entrainment)	Experimental laboratory setting	Preferred music listening reduced pain perception, while self-composed “pain” music increased pain ratings; entrainment methods modulated pain processing
Hajek et al. (2025) [36]	Germany	National observational study	Adults ≥80 years	Multimodal artistic activity (singing, music, visual arts, photography)	Community + institutional	Reduced loneliness, increased life satisfaction (context- and sex-dependent effects)

At a broader level, systematic reviews and meta-analyses support the role of performing arts in enhancing health-related quality of life, mental health, and functional outcomes across diverse populations [10,32-34]. Neurophysiological evidence further suggests that music and dance engage widespread brain networks, including effects on neuronal oscillations and functional connectivity [35]. Broader engagement in creative and artistic activities has also been associated with improved psychosocial well-being in older adults, supporting the role of arts-based participation across domains [36].

Visual arts, design and craft (n = 13): This category included modalities such as painting, pottery, embroidery, knitting, and jewellery-making, with painting and pottery being the most frequently studied. Methodologies ranged from randomized controlled trials to observational and qualitative designs.

In healthy populations, participation in visual arts and crafts was associated with increased happiness, greater life satisfaction, and reduced stress [36-39]. Embroidery-based interventions, in particular, were linked to decreased loneliness and enhanced social connectedness (Table 3) [40].

**Table 3:** Primary studies evaluating efficacy of Visual Arts, Design, and Craft in healthy populations

Author (Year)	Country	Study Design	Study Population	NeuroArts Modality	Setting	Outcomes
Hajek et al. (2025) [36]	Germany	National observational study	Adults ≥80 years	Multimodal artistic activity (singing, music, visual arts, photography)	Community + institutional	Reduced loneliness, increased life satisfaction (context- and sex-dependent effects)
Keyes et al. (2024) [37]	United Kingdom	Observational	General adult population	Creative arts & crafting	Community	Increase in life satisfaction, happiness, sense of purpose (independent of sociodemographics)
Chen & Buckingham (2025) [38]	New Zealand	Qualitative	Older Chinese migrants	Multimodal (gardening, visual arts, crafts, writing)	Community	Decrease in stress, improved adaptation, sustained well-being, identity integration
Gaspar da Silva 2023 [39]	Canada	Qualitative	Portuguese-speaking immigrant women	Storytelling + embroidery (art therapy	Virtual group	Increase in psychological well-being, social reconnection, cultural expression, identity reconstruction

In clinical populations, visual arts interventions demonstrated benefits across a variety of conditions. These included reductions in anxiety among individuals with mental health disorders, post-traumatic stress disorder (PTSD), type 1 diabetes, and trauma exposure [41-44]. Visual arts engagement was also associated with decreased depressive symptoms and reduced hopelessness in populations with depression, chronic stroke, and other mental health conditions [41,45-47]. Among older adults with mild cognitive impairment, visual arts interventions were associated with improvements in cognitive function, memory, executive functioning, and activities of daily living [47]. In healthcare settings, the integration of visual art within clinical environments was linked to higher patient satisfaction, improved patient-doctor communication, and enhanced staff well-being (Table 4) [48].

**Table 4:** Primary studies evaluating efficacy of Visual Arts, Design, and Craft in clinical populations ADL: Activities of Daily Living

Author (Year)	Country	Study Design	Study Population	NeuroArts Modality	Setting	Outcomes
Caddy et al. (2012) [40]	Australia	Observational	Psychiatric inpatients	Visual arts (painting/creative activity group)	Clinical (psychiatric hospital)	Participation in creative arts was associated with significant improvements in mental health outcomes (self-reported and clinician-rated)
Kimport & Hartzell (2015) [41]	United States	Observational	Psychiatric inpatients	Visual/tactile arts (clay art therapy)	Clinical (psychiatric unit)	Clay-based art therapy significantly reduced anxiety levels, with greater reductions observed in male participants
Gocen & Ozturk (2025) [42]	Turkey	Randomized Controlled Trial	Adolescents with type 1 diabetes	Visual arts (drawing, mandala, Zentangle)	Clinical (hybrid/remote delivery)	Art therapy significantly reduced anxiety and improved psychological well-being compared to control.
Özkafacı & Eren (2020) [43]	Turkey	Quasi-experimental	Female survivors of domestic violence with post-traumatic stress disorder	Visual arts (marbling art therapy)	Clinical/therapeutic	Art psychotherapy significantly reduced depression, anxiety, and hopelessness, and facilitated the expression of traumatic experiences
Haynes (2015) [44]	United States	Qualitative	Female victims of sex trafficking	Tactile art therapy (clay; group-based)	Community/therapeutic	Decrease in trauma symptoms, Increased empowerment and community building, improved life skills
Nan & Ho (2017) [45]	China	Randomized controlled trial	Adults with major depressive disorder	Tactile visual arts (clay art therapy)	Clinical (outpatient)	Clay art therapy significantly improved depressive symptoms, general health, emotional regulation, and well-being compared to visual art control
Yazici et al. (2024) [46]	Turkey	Randomized controlled trial	Chronic stroke patients	Tactile visual arts (clay therapy)	Clinical (rehabilitation)	Clay therapy + physical therapy significantly reduced depression and hopelessness compared to physical therapy alone
Zhao et al. (2018) [47]	China	Randomized controlled trial	Older adults with mild cognitive impairment (n=93)	Multimodal creative expression (art-based cognitive therapy)	Clinical/community	Creative expression therapy significantly improved global cognition, memory, executive function, ADLs, and maintained benefits at 6-month follow-up
Rice et al. (2008) [48]	United Kingdom	Comparative	Primary care patients and staff	Environmental arts (visual art, architectural design, healing environment)	Clinical (primary care)	Improved patient satisfaction, reduced anxiety, improved patient–doctor communication, and increased staff satisfaction in an enhanced environment with art and design elements

Literature (n = 12): The literature category encompassed writing, reading, and storytelling interventions, with writing-based approaches being the most commonly studied. Randomized controlled trials constituted a significant proportion of the included studies.

In healthy populations, literature-based interventions were associated with improved psychological outcomes. Creative writing interventions were linked to better psychological adjustment, reduced perceived stress, and decreased symptoms of depression and anxiety [49]. Early engagement in reading for pleasure was associated with improved cognitive performance and fewer mental health problems (Table 5) [50].

**Table 5:** Primary studies evaluating efficacy of Literature in healthy populations

Author (Year)	Country	Study Design	Study Population	NeuroArts Modality	Setting	Outcomes
Zhang et al. (2023) [49]	United States	Randomized Controlled Trial	Adults (132)	Narrative arts (expressive writing)	Community/experimental	Expressive writing improved mental health, reduced stress, depression, anxiety, and enhanced coping processes
Sun et al. (2024) [50]	United States	Observational	Children/adolescents	Narrative arts (reading for pleasure)	Community	Reading for pleasure was associated with improved cognitive performance, better mental well-being, and increased brain volume in multiple cortical regions

In clinical populations, findings were more variable. Some studies found no significant differences in quality of life or psychological outcomes following expressive writing interventions [51]. Creative writing interventions in patients with cancer were associated with improvements in mood and emotional well-being [52-54]. In certain cases, declines in quality of life over time were observed, likely reflecting disease progression and treatment burden [51]. In palliative care settings, poetry-based interventions were associated with reduced symptom burden and improved physician-patient communication [55]. Narrative-based approaches were linked to improved self-management and psychosocial outcomes in individuals with chronic disease [56]. Creative writing has also been explored in psychosocial rehabilitation among individuals with severe mental illness, demonstrating potential benefits in emotional regulation and recovery [57-59]. Digital storytelling also emerged as a promising modality for fostering empathy, self-expression, and insight into lived experiences, particularly in mental health contexts (Table 6) [60].

**Table 6:** Primary studies evaluating efficacy of Literature in clinical populations

Author (Year)	Country	Study Design	Study Population	NeuroArts Modality	Setting	Outcomes
Wu et al. (2020) [51]	China	Randomized Controlled Trial	Breast cancer patients undergoing chemotherapy	Narrative arts (expressive writing)	Clinical	No significant improvement in quality of life or psychological outcomes; intervention dosage did not affect outcomes
Nesterova et al. (2022) [52]	United States	Randomized Controlled Trial	Adult patients with cancer	Narrative arts	Clinical/community	Group creative writing improved mood (distress, anxiety, depression, anger), though no significant changes were seen in standardized depression/anxiety scales
Wright et al. (2024) [53]	United Kingdom	Randomized Controlled Trial	Children diagnosed with Autism	Narrative arts (Social Stories)	School/community	No significant improvement in social responsiveness, anxiety, or overall mental health; modest improvements in goal-specific behaviors and cost-effectiveness
Zhu et al. (2020) [54]	United States	Randomized Controlled trial	Patients with cancer	Narrative arts (group creative writing)	Clinical/community	Creative writing workshops were feasible and showed trends toward improved mood (reduced distress scores)
Segar et al. (2021) [55]	United States	Case-series	Palliative care patients	Narrative/literary arts (poetry reading)	Clinical (palliative care)	Poetry facilitated emotional expression, life reflection, improved patient–provider connection, and enhanced meaning-making
Goddu et al. (2015) [56]	United States	Qualitative study using in-depth interviews and focus groups	Adults with Diabetes mellitus (36)	Narrative arts (storytelling, role-play, film-based narratives)	Community	Narrative interventions improved self-efficacy, knowledge, attitudes, and facilitated behavior change through social support and storytelling
Dingle et al. (2017) [57]	Australia	Quasi-experimental	Adults with chronic mental health conditions (39)	Multimodal (choir singing + creative writing)	Community/group	Arts-based participation increased positive emotions and reduced negative emotions, with sustained reduction in negative effect and improved emotion regulation
Zachariae & O’Toole (2015) [58]	Denmark	Systematic review & Meta-analysis	Adult cancer patients or survivors	Narrative arts (expressive writing)	Clinical	No significant effects on psychological, physical, or quality-of-life outcomes; possible benefit only in subgroups with low emotional support
King et al. (2013) [59]	Australia	Qualitative	Individuals with severe mental illness	Narrative arts (creative writing)	Community/rehabilitation	Creative writing supported recovery processes, was well-accepted, and contributed to psychosocial rehabilitation
De Vecchi et al. (2016) [60]	Australia	Scoping review	Individuals in mental health contexts	Narrative arts (digital storytelling)	Community/mental health services	Digital storytelling facilitated sharing of lived experiences, enhanced empathy, learning, and mutual understanding; evidence base remains limited

Culture (n = 7): The cultural arts category primarily involved museum-based engagement, including visits to museums and galleries, as well as participation in structured museum programs. Study designs were heterogeneous, encompassing observational, qualitative, cohort, experimental, and systematic review methodologies.

In healthy populations, cultural engagement was associated with increased self-esteem, strengthened identity, reduced anxiety, enhanced well-being, improved cognitive reserve, and a greater sense of belonging and purpose (Table 7) [61-64].

**Table 7:** Primary studies evaluating efficacy of Culture in healthy populations

Author (Year)	Country	Study Design	Study Population	NeuroArts Modality	Setting	Outcomes
Binnie (2010) [61]	United Kingdom	Observational	Museum visitors and staff	Visual arts (museum art viewing)	Community (museum)	Viewing art in a museum setting was associated with reductions in anxiety and improvements in subjective well-being
Fancourt et al. (2018) [62]	United Kingdom	Longitudinal cohort study	Adults ≥50 years	Cultural engagement (museum attendance)	Community	Regular museum attendance was associated with a lower incidence of dementia over 10 years
Šveb Dragija & Jelinčić (2022) [63]	Croatia	Systematic review	Museum visitors	Passive visual arts (museum experience)	Community (museum)	Museum experiences were associated with enhanced psychological well-being; design factors (comfort, participation, engagement) influenced outcomes
Wallis & Noble (2022) [64]	United Kingdom	Qualitative (grounded theory, multimodal data)	Young children	Visual arts (museum/gallery interaction)	Community (museum)	Engagement with art spaces facilitated a sense of belonging, identity formation, and meaning-making through interaction with the environment

In clinical populations, museum-based interventions demonstrated multiple benefits. Engagement with museum objects was associated with increased self-confidence, facilitated reminiscence, and enhanced emotional processing among hospitalized patients [65]. In individuals with mild-to-moderate cognitive impairment, museum visits were linked to improvements in mood, well-being, personhood, cognition, and engagement [66]. A clinical-community intervention involving physician-prescribed museum visits also demonstrated significant improvements in mental well-being and quality of life (Table 8) [67].

**Table 8:** Primary studies evaluating efficacy of Culture in clinical populations

Author (Year)	Country	Study Design	Study Population	NeuroArts Modality	Setting	Outcomes
Chatterjee et al. (2009) [65]	United Kingdom	Mixed methods	Hospitalized patients (including the elderly)	Tactile arts (handling museum objects)	Clinical (hospital)	Handling museum objects improved life satisfaction, health perception, facilitated reminiscence, identity restoration, and emotional processing
Zeilig et al. (2022) [66]	United Kingdom	Systematic review	Individuals with mild-to-moderate dementia	Passive–interactive visual arts (museum-based programmes)	Community/museum	Museum-based programmes improved mood, well-being, cognition, engagement, and social outcomes in people with dementia
Beauchet et al. (2025) [67]	Canada	Experimental	Adult patients	Passive visual arts (physician-prescribed museum visit)	Clinical–community interface	Museum visits significantly improved well-being and quality of life; supports integration into healthcare pathways

Online, digital and electronic arts (n = 3): This category included digital visual art exposure and video-based creative expression.

In healthy populations, brief exposure to online art and cultural content was associated with improved mood, enhanced subjective well-being, and reductions in anxiety and loneliness (Table 9) [68].

**Table 9:** Primary studies evaluating efficacy of Online, Digital, and Electronic Arts in healthy populations

Author (Year)	Country	Study Design	Study Population	NeuroArts Modality	Setting	Outcomes
Trupp et al. (2022) [68]	Austria	Experimental	Adults	Digital visual arts	Digital/online	Brief exposure to online art improved mood, reduced anxiety and loneliness, and enhanced subjective well-being

In clinical populations, digital art interventions demonstrated promising outcomes. Exposure to digital and new media art was associated with improved quality of life and psychological well-being among patients undergoing intensive treatments such as stem cell transplantation [69]. Additionally, digital storytelling through vlogging was linked to reduced self-stigma and enhanced psychosocial recovery in individuals with mental health disorders (Table 10) [70].

**Table 10:** Primary studies evaluating efficacy of Online, Digital, and Electronic Arts in clinical populations

Author (Year)	Country	Study Design	Study Population	NeuroArts Modality	Setting	Outcomes
McCabe et al. (2013) [69]	Ireland	Randomized controlled trial	Patients undergoing stem cell transplantation	Digital visual arts (virtual window / new media art)	Clinical (hospital)	Digital art intervention reduced anxiety and depression and improved quality of life and patient experience
Sangeorzan et al. (2019) [70]	United Kingdom	Qualitative	Individuals with severe mental illness (YouTube vloggers)	Digital narrative arts (vlogging)	Digital/online	Vlogging facilitated peer support, reduced isolation and stigma, enhanced self-efficacy, and supported recovery processes

Discussion

Comparison With Previous Reviews

The findings of the present scoping review are broadly consistent with prior literature demonstrating beneficial associations between arts engagement and health outcomes. A landmark scoping review by Fancourt and Finn for the World Health Organization identified extensive evidence supporting the role of the arts in promoting physical and mental health across prevention, treatment, and rehabilitation settings [3]. Similarly, Stuckey and Nobel highlighted the relationship between artistic engagement, emotional expression, stress reduction, and public health outcomes across multiple artistic modalities [2].

However, the present review differs from earlier reviews in several important ways. First, rather than focusing solely on arts in health broadly, this review specifically examined the emerging field of NeuroArts and attempted to clarify its conceptual boundaries relative to related disciplines such as neuroaesthetics, empirical aesthetics, arts in health, and art therapy. Second, this review incorporated a wider range of contemporary modalities, including digital storytelling, virtual art exposure, online cultural engagement, and electronic arts, reflecting the expanding technological dimensions of NeuroArts research.

Compared with prior reviews that frequently concentrated on single modalities or specific populations, the present review synthesized evidence across multiple artistic domains and both healthy and clinical populations. This broader synthesis demonstrated that beneficial outcomes associated with NeuroArts extend beyond psychological well-being to include cognitive, social, behavioral, and quality-of-life outcomes across diverse settings.

In addition, the current review identified substantial heterogeneity in study designs, intervention characteristics, and outcome measures, consistent with observations from previous reviews [2,3]. Similar to prior literature, randomized controlled trials represented only a subset of the evidence base, and methodological variability remains a major limitation within the field. Nonetheless, the increasing number of experimental and interdisciplinary studies identified in this review suggests continued maturation of NeuroArts research as an emerging scientific domain.

Finally, while previous reviews primarily emphasized therapeutic and rehabilitative applications of the arts, the present review also highlights the potential role of NeuroArts in health promotion, disease prevention, cognitive resilience, and community well-being. This broader conceptual framing supports the growing view of NeuroArts as a multidisciplinary field with applications extending beyond traditional clinical intervention models.

Defining NeuroArts and Related Terminologies

NeuroArts has been described in multiple ways across the literature, reflecting its interdisciplinary scope and ongoing conceptual development. One of the earlier formulations, proposed in 2015, characterized NeuroArts as a field that integrates insights from the neuroscience of creativity, art, and neuroaesthetics to provide new perspectives on the creative functions of the brain [71].

Subsequently, the NeuroArts Blueprint Initiative in 2019 defined NeuroArts as a transdisciplinary field that examines how engagement with the arts and aesthetic experiences produces measurable effects on the brain, body, and behavior, and how such knowledge may be translated into interventions that enhance health and well-being [72]. This conceptualization has been reinforced in more recent literature, including a 2023 publication that similarly emphasized measurable biological and behavioral outcomes and their application to health-related practices [1].

More recent perspectives have further broadened the definition of NeuroArts. A 2024 study described it as a field concerned with the relationship between brain function and creativity across both healthy individuals and disease states [73]. Another contemporary definition highlights NeuroArts as an emerging scientific domain investigating how diverse art forms - such as music, visual arts, dance, and architecture - can promote health and well-being through multisensory engagement and therapeutic applications [74].

Drawing from these existing definitions and the findings of the present review, we propose a unified definition of NeuroArts as:

“a field of science that examines how experiences derived from creativity, arts, and aesthetics produce measurable neural, physiological, and behavioral changes, and how these changes influence health and well-being.”

To further delineate NeuroArts from related disciplines, it is important to clarify several adjacent concepts, including neuroaesthetics, empirical aesthetics, arts in health, and art therapy.

Neuroaesthetics

Neuroaesthetics was first formally introduced by Semir Zeki in 1999 as a field focused on understanding the neurobiological mechanisms underlying aesthetic perception, production, judgment, and experience. It has since evolved into an interdisciplinary domain that examines the neural processes involved in aesthetic experiences and related perceptual phenomena [75,76].

Empirical Aesthetics

Empirical aesthetics is a research area situated at the intersection of psychology and neuroscience that investigates how individuals perceive, evaluate, and generate aesthetic experiences [77]. Within this domain, a distinction has been made between the cognitive science of aesthetics - concerned with sensory evaluation and aesthetic judgment beyond art-specific contexts - and the cognitive science of art, which focuses on the broader neurobiological processes involved in experiencing art [78].

While the cognitive science of aesthetics addresses how evaluative processes arise from sensory input, the cognitive science of art extends beyond this to examine how artworks engage neural systems related to emotion, cognition, and meaning-making [78].

Arts in Health and Art Therapy

Arts in Health refers to the use of artistic practices to support health and well-being across a wide range of settings, including both community and clinical contexts [79]. This approach encompasses diverse interventions and populations, including individuals without illness as well as those with medical conditions [2,3,79].

In contrast, art therapy is a structured clinical intervention that utilizes artistic media to facilitate self-expression and psychological processing under the supervision of a trained therapist [80,81]. It is primarily applied within clinical populations and is grounded in established therapeutic frameworks [80,81].

Synthesis of Terminologies

**Figure 3:**
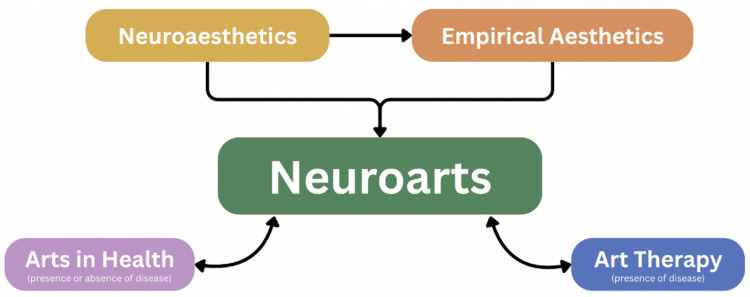
Conceptual Framework of NeuroArts and Related Terminologies

To clarify the relationships among these concepts, a conceptual framework was developed (Figure 3).

Within this framework, neuroaesthetics and empirical aesthetics represent broader scientific domains that investigate the neural and psychological foundations of aesthetic experience. NeuroArts can be understood as a related but more application-oriented field, with a particular emphasis on how these mechanisms translate into measurable health outcomes.

Arts in Health reflects the practical implementation of artistic interventions across populations, whereas art therapy represents a specialized clinical discipline focused on the therapeutic use of art within structured psychological contexts.

Implications for Policy, Practice and Further Research

The observed health outcomes across different art forms in both healthy and clinical populations highlight the potential of NeuroArts as both a health-promoting strategy and a complementary therapeutic modality across the health-disease continuum (Table 11) [2,3].

**Table 11:** Summary of Health Outcomes of NeuroArts in Healthy and Clinical Populations Based on Available Literature

Art Forms	Health Outcomes			
	Healthy populations		Clinical populations	
Performance Arts	Associated with improvements in global cognition, well-being, pain tolerance, and stress reduction		Associated with improvements in post-surgical recovery, quality of life in patients with chronic obstructive pulmonary disease (COPD) and those undergoing chemotherapy, cognitive function in patients with dementia, and motor function in patients with cerebral palsy and Parkinson’s disease	
Visual Arts, Design and Craft	Associated with improvements in well-being and reductions in stress		Associated with reductions in anxiety and depression in patients with mental health disorders and stroke, preservation of cognitive function in patients with dementia, and improved patient–doctor communication in outpatient settings	
Literature	Associated with improvements in mental health and cognitive function		Associated with improvements in mood in patients with cancer and reduced symptom burden in palliative care; no significant effect on social responsiveness and mood in patients with autism; decline in quality of life observed in patients undergoing chemotherapy	
Culture	Associated with improvements in well-being, reducing anxiety, and preservation of cognition		Associated with improvements in mood and cognition in patients with dementia and increased self-confidence among hospitalized patients	
Online, Digital and Electronic Arts	Associated with improvements in mood, well-being and overall life satisfaction		Associated with improvements in mental health and recovery in post-procedural patients and individuals with mental health disorders	

Performing arts emerged as the most extensively studied category, with music as the most frequently investigated modality. Music is deeply embedded in daily life and personal identity, often evoking autobiographical memories intertwined with emotional experiences. These effects likely arise from activation of distributed neural networks involving frontal, temporal, and sensorimotor regions, supporting emotion regulation, memory, and neuroplasticity [82].

The observed benefits of music on mood regulation, pain tolerance, and stress reduction may therefore reflect integrated neurophysiological and psychological mechanisms. Additionally, the preservation of global cognition associated with music interventions may be explained by the engagement of overlapping cortical regions. These regions include the temporal gyri, frontal gyri, supramarginal gyrus, and precentral gyrus, which support learning, memory, and sensorimotor integration. Repeated activation of these networks may facilitate neuroplasticity and contribute to the preservation of neural pathways [82].

Dance was the second most commonly studied modality within performing arts. As a multimodal activity, dance integrates motor, cognitive, visuospatial, emotional, and social processes. Dance-based interventions were associated with improvements in motor coordination and balance and may contribute to the development of “motor reserve,” which has been hypothesized to provide protective effects against aging and neurodegenerative conditions [83].

Visual arts, design, and craft represented the second most studied category. Engagement in visual arts allows individuals to express symbolic and emotional content, facilitating insight, stress reduction, and trauma processing. These creative processes have been associated with improvements in emotional regulation, cognitive function, and interpersonal relationships, ultimately contributing to enhanced well-being and self-fulfillment [84].

In clinical populations, visual arts interventions have demonstrated benefits in reducing symptoms of depression and anxiety, particularly in patients with mood disorders. In individuals with mild cognitive impairment, visual arts have also been applied as a form of cognitive rehabilitation, with reported improvements in cognitive function, memory, executive functioning, and activities of daily living [45-48].

Literature-based interventions constituted the third most studied category. From a neurodevelopmental perspective, reading engages distributed neural pathways, including dorsal temporo-parietal circuits and ventral occipito-temporal regions, with increasing specialization and lateralization over time [85]. Beyond these structural mechanisms, literary engagement influences cognition through processes such as inference generation, mental simulation, imagery, and narrative immersion, which collectively contribute to emotional and cognitive enrichment [85].

Cultural engagement, particularly museum-based activities, remains an underutilized but promising intervention. Emerging evidence suggests that even a single physician-prescribed museum visit can lead to measurable improvements in mental well-being and quality of life [67].

The most recent and least studied category was online, digital, and electronic arts. Digital platforms offer accessible and scalable avenues for artistic engagement. Similar to traditional visual arts, digital modalities provide opportunities for self-expression and identity formation. Early evidence suggests that digital art exposure and creation may improve psychological well-being and support recovery processes, particularly in individuals with mental health conditions [68,70].

The findings of this review suggest that arts-based interventions may be valuable complementary approaches in both clinical and community settings. For practice, integrating creative activities into therapeutic environments may enhance patient engagement and well-being. For policy, there is a need to support interdisciplinary approaches that incorporate the arts into health systems. Future research should focus on standardizing methodologies, exploring underrepresented art forms, and investigating the mechanisms underlying observed benefits.

Gaps and Future Directions for Research

This review provides a foundational roadmap for the field of NeuroArts and highlights several key areas for future investigation. While current evidence supports the therapeutic and health-promoting effects of art, the overall evidence base remains limited by variability in study design, population characteristics, and outcome measures [2,3].

Future research should prioritize the development of standardized methodologies and outcome measures to enable comparability across studies. Larger, well-designed trials across diverse populations, including variations in age, sex, cultural background, and disease states are needed to strengthen the generalizability of findings.

Additional research into specific modalities, such as different genres of music, textile-based arts (e.g., embroidery), and emerging digital forms, is warranted. Additionally, research should expand beyond traditionally studied populations, particularly mental health and neurological conditions, to explore applications in other medical and preventive contexts.

Importantly, NeuroArts should also be examined as a strategy for disease prevention and health promotion, not solely as a therapeutic intervention. Advancing this field will require a deeper understanding of underlying mechanisms, as well as the development of evidence-based frameworks for integrating NeuroArts into clinical practice and public health systems.

Limitations of the study

A key strength of this review is the use of broad search criteria and an extensive literature search spanning over 25 years, allowing for the inclusion of diverse study designs, populations, and artistic modalities. This comprehensive approach enabled a more holistic understanding of NeuroArts and its applications across both healthy and clinical populations.

However, several limitations should be acknowledged. There was substantial heterogeneity among included studies in terms of interventions, populations, and outcome measures, which limits direct comparability and synthesis of findings. Although many studies employed randomized controlled or quasi-experimental designs, variability in methodological quality may affect the robustness of conclusions.

In addition, this review was limited to peer-reviewed literature and may not capture unpublished or community-based interventions that are not formally documented. Finally, the evolving nature of NeuroArts means that some modalities, particularly digital and technology-based interventions, remain underrepresented in the current evidence base.

## Conclusions

This scoping review presents a comprehensive synthesis of NeuroArts, tracing its development from early conceptualizations to its current applications across diverse populations. Based on the findings of this review, NeuroArts may be defined as a field of science that examines how experiences derived from creativity, the arts, and aesthetics lead to measurable neural, physiological, and behavioral changes, and how these changes contribute to health and well-being. This study also distinguishes NeuroArts from related domains, including neuroaesthetics, empirical aesthetics, arts in health, and art therapy, thereby clarifying its conceptual boundaries within the broader scientific and clinical landscape.

The evidence synthesized in this review indicates that a range of artistic modalities are associated with beneficial outcomes in both healthy and clinical populations. These include improvements in psychological well-being, cognitive performance, and overall quality of life. Collectively, these findings support the role of NeuroArts as a complementary approach to health promotion and disease management. Despite these promising findings, the current body of evidence remains heterogeneous in terms of study design, populations, and outcome measures. Future research should prioritize the use of standardized methodologies, inclusion of larger and more diverse populations, and the application of rigorous experimental designs to further strengthen the evidence base. As the field continues to advance, NeuroArts has the potential to serve as an integrative and scalable approach to improving health and well-being across the lifespan.

## Appendices

**Table 12:** Representative PubMed Search Strategy

Search Step	Search Terms	Records Identified
1	(singing OR dancing OR musical instrument OR drama OR performing in a play) AND (effect on health)	2,456
2	(painting OR sculpture OR drawing OR textile OR crafts) AND (effect on health)	1,096
3	(reading for pleasure OR creative writing OR composing music OR stories OR storytelling) AND (effect on health)	3,926
4	(community arts OR cultural festival OR fairs OR cultural events OR museums OR galleries OR theatre OR concerts OR exhibitions) AND (effect on health)	952
5	(digital artworks OR computer animations OR film-making OR videos OR photography OR radio plays OR television dramas OR vlogging) AND (effect on health)	889

**Table 13:** Performing Arts in Healthy Populations

Study	Population & Setting	NeuroArts Intervention	Outcomes
LiKamWa et al. [4]	Healthy adults recruited in Gainesville, Florida, USA (N=40; 23 females, 17 males; mean age 26.6 ± 7.8 years; age range 18–45 years) in a quasi-experimental university/community-based research setting	Music-based intervention involving three experimental conditions: silence, passive music listening, and active singing using participant-selected songs. Participants underwent cold pressor quantitative sensory testing during each condition. Singing condition involved active singing along with audiovisual lyric display. Conditions were randomized with 10-minute washout periods between sessions. Intervention supervised by the study research team at the University of Florida. Total experimental session duration approximately 60 minutes.	Singing significantly increased cold pain threshold and tolerance compared with listening and silence conditions. Significant differences were observed for pain threshold (χ²(2)=10.1974, p=0.0061) and tolerance time (χ²(2)=24.4696, p<0.0001). Singing vs silence: p<0.0001; singing vs listening: p=0.0008. Greater self-reported singing proficiency correlated with higher pain tolerance (r=0.40, p=0.015). Participants also demonstrated higher positive affect scores after singing.
Wulff et al. [5]	Prospective randomized controlled trial involving 172 pregnant women (aged 18–42 years; mean age 34.03 ± 3.89 years) recruited from the Clinic for Gynecology and Obstetrics at Heinrich-Heine-University Düsseldorf, Germany. Participants were enrolled between 24+0 and 35+6 weeks gestation and randomized into singing, music-listening, or control groups	Prenatal music and singing intervention conducted between approximately the 30th and 36th gestational weeks. The music group participated in weekly 30-minute guided passive music-listening relaxation sessions using calm classical music and home-based daily listening (10–15 minutes/day). The singing group attended 2–4 group singing sessions led by a music therapist, involving live guitar-accompanied lullabies and children’s songs, followed by prescribed daily home singing practice (10–15 minutes/day). Salivary cortisol, alpha-amylase, and oxytocin samples were collected before and after intervention sessions.	Both music and singing interventions significantly improved emotional state, reduced salivary cortisol, and increased oxytocin levels immediately after intervention sessions. Singing produced significantly greater reductions in cortisol compared with passive music listening (interaction: F(1,114)=12.02, p=0.001). Significant improvements were also observed in perceived closeness to the unborn child and maternal self-efficacy, particularly in the singing group. No significant effects were observed for depressive symptoms or MAAS bonding questionnaire scores.
de Witte et al. [6]	Systematic review and multilevel meta-analysis including 47 studies, 76 effect sizes, and 2,747 participants from both medical and mental healthcare settings across clinical and community populations. Included studies comprised randomized controlled trials (RCTs) and clinical controlled trials (CCTs) evaluating stress-related outcomes in diverse populations.	Personalized music therapy interventions delivered by trained music therapists, including active and receptive modalities such as live music, prerecorded music, improvisation, singing, relaxation-based listening, and therapist-guided interactive music engagement. Interventions were individualized according to patient needs and preferences, distinguishing music therapy from passive “music medicine” approaches. Moderator analyses examined session frequency, intervention duration, delivery format, and intervention protocols.	Music therapy demonstrated a significant medium-to-large overall effect on stress-related outcomes (d = 0.723; 95% CI 0.51–0.94), including reductions in psychological stress, anxiety, and physiological arousal measures such as heart rate, blood pressure, and stress hormone levels. Larger effects were observed in clinical controlled trials compared with RCTs, in waiting-list control conditions, and in studies conducted in non-Western countries. Findings support music therapy as an effective non-pharmacologic intervention for stress reduction across diverse healthcare settings.
Tunçgenç et al. [7]	Randomized controlled longitudinal study involving 58 adolescents aged 11–16 years in the United Kingdom during the COVID-19 pandemic. Participants were randomly assigned to either an intervention group or waitlist control group. Most participants were female (52 girls), with diverse ethnic representation.	A 5-week online group hip-hop dance program delivered via Zoom by professional dance instructors. Weekly 1-hour sessions included warm-ups, choreography, creative movement tasks, breakout room interactions, synchronized group dancing, and collaborative movement exercises designed to foster social connection and emotional expression.	Adolescents participating in the dance intervention demonstrated significantly greater improvements in social bonding (p < .0001), well-being (p < .0001), and hope/future orientation compared with waitlist controls. Increased social bonding significantly predicted improvements in well-being, while improvements in both bonding and well-being predicted a stronger future outlook and hopefulness. The study concluded that online group dance interventions can strengthen social connectedness and mental well-being even during periods of social isolation.
Wind et al. [8]	Pilot EEG study involving 11 healthy dance-inexperienced female participants (mean age 24.3 years) from Johannes Gutenberg-University Mainz, Germany. Participants underwent a 3-week Modern Jazz Dance training program prior to EEG testing.	Participants learned and performed a Modern Jazz Dance (MJD) choreography under four randomized conditions: (1) physically executed dance with music, (2) physically executed dance without music, (3) imagined dance with music, and (4) imagined dance without music. EEG recordings before and after each condition evaluated electrical brain activity and functional connectivity across theta, alpha, beta, and gamma frequency bands.	Physically executed dance produced significantly greater increases in brain activity and functional connectivity compared with imagined dance, particularly in alpha and beta frequency bands. Dance without music generated the strongest increases in frontal, central, temporal, and parietal alpha activity, suggesting enhanced attentional processing, motor planning, spatial cognition, memory engagement, and relaxed wakefulness. EEG connectivity findings also demonstrated increased fronto-occipital and frontal-temporal coherence associated with executive function and sensorimotor integration. In contrast, imagined dance without music led predominantly to decreased brain activity and connectivity across multiple frequency bands. The study concluded that physically executed dance induces widespread neurophysiological activation and functional network integration that may underlie the cognitive, emotional, and therapeutic benefits associated with dance interventions.
Agres and Chen [9]	Randomized controlled trial involving 76 university students from the National University of Singapore (mean age 22.6 years; 63 females, 12 males). Participants were randomized into an Experimental group (Movin’ & Groovin’ for Wellness [MGW]) and a Control group over a 10-week term-period intervention.	The MGW program was a 10-week participatory performing arts intervention integrating facilitated group drumming, dance, movement improvisation, and live music. Participants attended weekly 1.5-hour sessions focused on creative expression, emotional processing, social interaction, and non-verbal communication. Students alternated between drumming and dance modules across the intervention period.	The intervention produced significant improvements in mental health, quality of life, stress reduction, creativity, and social connection compared with controls. Experimental participants demonstrated significant reductions in DASS-21 (Depression, Anxiety, and Stress Scale - 21 items) stress scores by Week 10 and improvements in WHOQOL-BREF (World Health Organization Quality of Life – Brief Version) psychological and physical health domains. Creativity scores (Experience of Creation Scale) significantly increased at Weeks 5 and 10 with moderate-to-large effect sizes. Qualitative findings revealed enhanced mood, emotional expression, social bonding, and formation of meaningful friendships. Participants described the program as providing a psychologically safe, stigma-free environment that facilitated emotional release, self-expression, social cohesion, and stress coping through music and movement. The study concluded that combined drumming, dance, and improvisation-based NeuroArts interventions can effectively support student mental health and well-being in culturally appropriate and socially engaging ways.
Hwang and Braun [10]	Systematic literature review of 18 studies involving older adults aged 52–87 years across North America, South America, Europe, and Asia. Included both healthy older adults and older adults with clinical conditions such as visual impairment and metabolic syndrome. Most studies predominantly involved female participants, with several studies including exclusively female cohorts. Interventions were conducted in community, rehabilitation, institutional, and outpatient settings.	Dance interventions included ballroom (e.g., tango, salsa, foxtrot, bolero, swing, waltz, cha-cha), contemporary dance, improvisational dance, jazz dance, and culturally specific dance forms (e.g., Greek, Turkish, Korean, Cantonese, and line dancing). Intervention frequency ranged from 1–4 sessions per week, with program durations ranging from 6 weeks to 8 months and session durations ranging from 45 minutes to 2 hours. Interventions were delivered in structured group settings and included dance therapy, recreational dance, and culturally adapted movement programs.	Dance interventions demonstrated significant improvements across multiple physical and cognitive domains in older adults. Positive findings were reported in flexibility (3/5 measures, 60%), muscular strength and endurance (23/28 measures, 82%), balance (8/9 measures, 89%), cardiovascular endurance, and cognitive function (8/10 measures, 80%). Improvements were particularly notable in functional fitness, postural stability, mobility, and cognition. Studies involving older adults with visual impairment and metabolic syndrome also demonstrated beneficial outcomes. Attrition rates were generally low among studies reporting adherence. The review concluded that dance interventions, regardless of style, can significantly improve physical health, balance, cognition, and functional independence in older adults.

**Table 14:** Performing Arts in Clinical Populations MMSE: Mini-Mental State Examination

Study	Population & Setting	NeuroArts Intervention	Outcomes
Vetter et al. [11]	Systematic review and meta-analysis of 48 studies evaluating art-based interventions among adult surgical patients across inpatient and outpatient perioperative settings in multiple countries. The review primarily included controlled trials involving patients undergoing various surgical procedures under general, regional, or local anesthesia. A total of 47 studies specifically evaluated perioperative music interventions, while 1 study assessed sunlight exposure in postoperative recovery.	NeuroArts interventions included perioperative music exposure before, during, and/or after surgery, architectural/environmental design features, natural sunlight exposure, visual art, room aesthetics, and ambient environmental modifications. Music interventions involved both self-selected and preselected music delivered within 24 hours of surgery. Architectural and visual interventions included nature imagery, spacious room design, increased sunlight exposure, color redesign, sound-absorbing ceilings, and healing-environment hospital architecture.	Meta-analysis demonstrated significant beneficial effects of perioperative music on postoperative pain (effect size [ES] = −0.31; 95% CI −0.46 to −0.17), pain medication use (ES = −0.28; 95% CI −0.45 to −0.11), anxiety (ES = −0.34; 95% CI −0.51 to −0.17), systolic blood pressure (ES = −0.40; 95% CI −0.59 to −0.21), and heart rate (ES = −0.27; 95% CI −0.44 to −0.10). Self-selected music produced greater reductions in pain and anxiety compared with preselected music. Additional narrative findings suggested that natural sunlight, spacious architectural design, nature imagery, ambient sound reduction, and aesthetically enhanced hospital environments may reduce stress, postoperative pain, analgesic requirements, and patient anxiety while improving satisfaction and perceived well-being.
Lima et al. [12]	Randomized nonblinded clinical trial involving 33 female breast cancer patients undergoing adjuvant chemotherapy with doxorubicin and cyclophosphamide (AC protocol) at two oncology referral hospitals in São Luis, Maranhão, Brazil (Aldenora Bello Hospital and São Domingos Hospital). Group Music (GM): n=16; Group Control (GC): n=17. Mean age: GM 49.5 ± 10.65 years; GC 50.76 ± 9.45 years. Participants had ECOG performance scores ≤2 and nonmetastatic disease. The intervention was conducted during the first three chemotherapy cycles in an outpatient chemotherapy setting.	Participants in the GM received a standardized 30-minute pre-chemotherapy music intervention delivered through MP3 headphones immediately before each chemotherapy session. The playlist was preselected by the investigators and consisted of relaxing classical music and movie soundtracks, including works by Yiruma, Schubert/Liszt, The Piano Guys, Sarah McLachlan, and the Titanic soundtrack music. Prior to music exposure, participants underwent guided breathing and self-relaxation exercises facilitated verbally by a researcher. Sessions were performed during each of the first three chemotherapy cycles. The GC received verbal self-relaxation instructions only without music exposure.	Music intervention was associated with significantly improved quality of life scores measured using the WHOQOL-BREF after the first and third chemotherapy sessions compared with controls (P = .04 and P = .02, respectively). Depression scores measured using the Beck Depression Inventory-II (BDI-II) were significantly lower in the GM at the final assessment (13.3 ± 4.2 vs 21.0 ± 6.4; P = .001). Anxiety scores measured using the Beck Anxiety Inventory (BAI) were also significantly lower in the GM at the final phase (5.9 ± 6.2 vs 20.1 ± 9.4; P = .001). The incidence of chemotherapy-related vomiting was significantly lower in the GM during the third chemotherapy cycle (P = .010). All participants in the intervention group reported positive subjective effects, including reduced stress, improved mood, increased relaxation, reduced fatigue, enhanced self-esteem, and better emotional well-being.
Nguyen et al. [13]	Pilot assessor-blinded randomized controlled trial involving 24 women with breast and gynecological cancer receiving chemotherapy at an oncology hospital in Hanoi, Vietnam. Intervention group: n=12; control group: n=12. Mean age was 52.29 ± 13.44 years (range 31–75 years). Breast cancer accounted for 79.2% of participants, while 20.8% had gynecological cancers. Approximately 45.8% had stage IV disease. Participants were receiving chemotherapy every three weeks and had at least three chemotherapy cycles remaining. The intervention was primarily home-based following an initial face-to-face training session conducted in a private room within the chemotherapy unit.	The intervention group received music intervention combined with progressive muscle relaxation (PMR). Participants first underwent face-to-face training conducted by the principal investigator, certified in PMR and music intervention. PMR involved sequential tensing and relaxation of 16 muscle groups, with each muscle group tightened for 5–7 seconds and relaxed for 20–30 seconds, repeated twice. Participants then listened to 20 minutes of selected instrumental relaxing music with slow rhythm and soft melodies (60–80 beats/minute), followed immediately after the PMR session. Music genres included Vietnamese folk songs, religious music, revolutionary music, and Vietnamese bolero instrumental tracks based on patient preference. Participants self-practiced the intervention once daily at home for 3 weeks using MP3 recordings sent to their mobile phones. Weekly follow-up calls and family support reinforcement were incorporated to improve adherence. The control group received standard oncology care, including routine health assessment, nutritional advice, and chemotherapy-related consultations.	Anxiety, depression, and stress were evaluated using the Depression Anxiety Stress Scale-21 (DASS-21), while quality of life was assessed using the Functional Assessment of Cancer Therapy–General (FACT-G). Greater reductions in anxiety were observed in the intervention group compared with controls at both week 3 (Hedges’ g = 0.57, 95% CI: -0.20 to 1.35) and week 6 follow-up (g = 0.52, 95% CI: -0.30 to 1.34). Depression scores likewise improved at week 3 (g = 0.21, 95% CI: -0.55 to 0.97) and week 6 (g = 0.60, 95% CI: -0.23 to 1.42). Stress levels showed modest reductions favoring the intervention group at week 3 (g = 0.32, 95% CI: -0.44 to 1.08). Quality of life improved in the intervention group compared with controls at week 3 (g = 0.43, 95% CI: -0.33 to 1.20) and week 6 (g = 0.82, 95% CI: -0.03 to 1.66). Qualitative interviews demonstrated perceived improvements in relaxation, sleep, pain, nausea, vomiting, fatigue, emotional comfort, and stress reduction, with participants expressing willingness to continue practicing the intervention in the future.
Philip et al. [14]	Pilot randomized controlled trial involving 18 adults with chronic obstructive pulmonary disease (COPD) recruited from a specialist COPD clinic at the Royal Brompton Hospital, London, United Kingdom. Participants were randomized to Singing for Lung Health (SLH) intervention (n=9) or usual care (n=9). Mean age was 72.1 ± 9.65 years in the SLH group and 69.89 ± 9.36 years in controls. Female participants comprised 33% of the SLH group and 66% of controls. The intervention was conducted initially in a face-to-face outpatient hospital setting before transitioning to online delivery via Zoom due to COVID-19 restrictions.	Singing for Lung Health (SLH) intervention consisted of 12 weekly 1-hour group singing sessions specifically designed for patients with chronic respiratory disease. Sessions included physical warm-ups, breathing exercises, vocal warm-ups, singing activities, and cool-down exercises. The first 5 sessions were conducted face-to-face at Royal Brompton Hospital, followed by 7 online sessions delivered via Zoom after a 2-week transition period related to the COVID-19 pandemic. Sessions were led by a professional singing teacher with 4 years of SLH experience. Participants also received CDs containing singing exercises and were encouraged to practice daily at home. Attendance was 90% during face-to-face sessions and 53% during online sessions.	Participants reported perceived improvements in breathing control, mood, social connectedness, exercise participation, and emotional well-being. Quantitative outcomes demonstrated significant improvements in depression scores measured using the Patient Health Questionnaire-9 (PHQ-9) (treatment effect −4.78 points, p<0.05; minimum clinically important difference [MCID] 5 points) and balance confidence measured using the Activity-specific Balance Confidence (ABC) Scale (treatment effect +17.21 points, p=0.04; MCID 14.2). Improvements in SF-36 health change scores were also observed (p=0.026). Qualitative thematic analysis identified perceived psychosocial benefits, reduced social isolation, enhanced mood, improved breathing control, and increased confidence. Barriers to online participation included limited digital literacy, internet access difficulties, and reduced interpersonal interaction compared with face-to-face sessions.
Lewis et al. [15]	Systematic review and consensus statement evaluating Singing for Lung Health (SLH) interventions among adults with chronic respiratory diseases, including chronic obstructive pulmonary disease (COPD), asthma, bronchiectasis, interstitial lung disease, and obstructive sleep apnea. Six studies were included (4 randomized controlled trials and 2 cohort studies) conducted in the United Kingdom, Brazil, Canada, and Australia. Across the included studies, participant ages ranged from approximately 29.7 to 71.7 years, with most cohorts composed predominantly of COPD patients. Interventions were delivered in outpatient hospital settings, pulmonary rehabilitation-associated community settings, community singing groups, and Indigenous community-based programs.	Singing for Lung Health (SLH) interventions consisted of structured singing classes specifically designed for individuals with respiratory disease. Sessions generally included physical warm-ups, breathing exercises, vocal warm-ups, singing exercises, posture training, song practice, and cool-down activities. Intervention duration varied across studies from 4 weeks to 36 weeks, with sessions conducted once or twice weekly, lasting 30–90 minutes. Some interventions incorporated breathing control instruction from physiotherapists, diaphragmatic breathing exercises, home practice using CDs, and community singing activities. Singing sessions were led by trained singing teachers, physiotherapists, or respiratory health professionals with expertise in respiratory-focused singing techniques.	Quantitative findings suggested that SLH may improve health-related quality of life, particularly physical health domains measured using the Short Form-36 (SF-36), and reduce anxiety symptoms without major adverse effects. One randomized controlled trial demonstrated significant improvement in SF-36 physical component scores (+7.5 vs. −3.8; p=0.02) and Hospital Anxiety and Depression Scale anxiety scores (−1.1 vs. +0.8; p=0.03). Another study showed greater improvement in SF-36 physical component scores after 8 weeks of singing classes (12.9 vs. −2.5; p=0.02). Additional reported benefits included increased maximal expiratory pressure, transient increases in inspiratory capacity, improved oxygen saturation, enhanced breath control, improved posture, greater confidence in managing breathlessness, and increased social connectedness. Qualitative findings consistently described singing as enjoyable, socially supportive, psychologically uplifting, and beneficial for coping with chronic respiratory disease. However, the review emphasized substantial heterogeneity across studies, small sample sizes, risk of bias, and the need for larger long-term trials.
Okur and Nural [16]	Randomized controlled study involving patients with chronic obstructive pulmonary disease (COPD). Detailed demographic characteristics and the study setting were not fully accessible from the available abstract.	Singing and melodica-playing interventions designed for patients with COPD. Detailed intervention duration, session frequency, facilitator information, and session structure were not fully available from the accessible source.	The intervention was reported to improve disease symptoms, self-efficacy, and exercise capacity among COPD patients. Detailed statistical outcomes were not accessible from the available source.
Gómez-Gallego et al. [17]	Quasi-experimental study involving 90 nursing home residents with mild-to-moderate Alzheimer’s disease (AD) recruited from six nursing homes in the Region of Murcia, Spain. Participants were assigned to active music intervention (AMI; n=28, 71.5% female, mean age 83.93 ± 8.01 years), receptive music intervention (RMI; n=21, 61.9% female, mean age 78.67 ± 5.73 years), or control activity (n=41, 54.5% female, mean age 80.02 ± 5.78 years). Approximately 70% had mild dementia and 30% had moderate dementia based on Clinical Dementia Rating scores. The intervention was conducted in nursing home community settings.	Active Music Intervention (AMI) and Receptive Music Intervention (RMI) were conducted twice weekly for 3 months (24 total sessions), with each session lasting approximately 45 minutes. AMI sessions included welcome songs, rhythmic hand-clapping exercises, dance and movement activities, music quizzes, singing, and farewell songs designed to improve cognition, socialization, movement fluency, and mood. RMI involved passive listening to participant-preferred music playlists while discussing emotions and memories associated with the songs. Sessions were facilitated by music therapists with master’s-level qualifications in creative arts therapy and specialization in music therapy. Preferred genres included boleros, sevillanas, and religious music. Control participants watched nature documentaries without music.	Active music intervention significantly improved cognition, neuropsychiatric symptoms, and functional abilities compared with receptive music intervention and control activities. MMSE scores significantly increased in the AMI group (p<0.001) with a large effect size (η² = 0.62). Neuropsychiatric Inventory (NPI) scores significantly decreased after AMI (p<0.001; η² = 0.61). Barthel Index functional scores improved significantly in the AMI group (p<0.001; η² = 0.18). Improvements in Tinetti Scale motor scores were observed but did not reach significance between groups (p=0.110). Receptive music intervention demonstrated stabilizing effects on neuropsychiatric symptoms compared with controls. No significant effects were observed for depressive symptoms measured using the Geriatric Depression Scale. The percentage of participants showing improvement in cognition (85.7%), behavioral symptoms (92.9%), and functional state (46.4%) was substantially higher in the AMI group compared with the RMI and control groups.
Bleibel et al. [18]	Systematic review of 8 randomized controlled trials involving 689 participants with Alzheimer’s disease (AD), including approximately 300 female participants (43.5%), with mean ages ranging from 60.47 to 87.1 years. Studies were conducted across Europe (France and Spain), Asia (China and Japan), and the United States. Included populations ranged from mild to severe AD dementia, with baseline MMSE scores ranging from 4.65 to 25.07. Interventions were conducted in outpatient, nursing home, and community-based dementia care settings.	Included studies evaluated both active music intervention (AMI) and receptive music intervention (RMI). AMI modalities included singing, dancing, rhythmic movement, clapping, instrument playing, music quizzes, and active participation in musical activities. RMI involved passive listening to individualized or familiar music playlists. Intervention durations ranged from 4 weeks to 3 months, with session frequencies varying from twice weekly to structured ongoing programs. Sessions were delivered by music therapists, choir conductors, musicians, or trained facilitators, depending on the study. Many interventions used individualized playlists tailored to patient preferences and autobiographical memories.	Seven of eight included randomized controlled trials demonstrated significant cognitive or neuropsychiatric benefits associated with music therapy interventions in AD patients. Reported improvements included global cognition (MMSE, MoCA), memory (Free and Cued Recall Test [FCRT], WHO-UCLA Auditory Verbal Learning Test [WHO-UCLA AVLT]), executive function (Trail Making Test [TMT]), verbal fluency, attention, psychomotor speed, and neuropsychiatric symptoms. Active music interventions generally demonstrated superior outcomes compared with receptive interventions. Reported findings included significant improvements in MMSE scores (p<0.001), memory functioning (p=0.007), executive function (p=0.006), psychomotor speed and attention (p=0.04), and verbal memory stabilization (p=0.001). The review concluded that music therapy may serve as an effective complementary non-pharmacologic intervention for improving cognitive and behavioral symptoms in Alzheimer’s disease, although heterogeneity among studies and intervention methods was substantial.
Liu et al. [19]	Systematic review and meta-analysis of 6 studies involving 115 patients with post-stroke aphasia undergoing rehabilitation in clinical and neurorehabilitation settings. Included studies evaluated patients with language impairment following ischemic or hemorrhagic stroke across hospital-based rehabilitation and outpatient therapy programs.	Music therapy interventions included melodic intonation therapy, rhythmic speech therapy, singing-based language exercises, structured vocalization, and music-supported language rehabilitation techniques delivered alongside conventional speech therapy. Interventions were designed to stimulate language recovery through rhythm, melody, vocal repetition, and musical cueing. Therapy duration and frequency varied across included studies. Sessions were generally administered by music therapists, rehabilitation specialists, or speech-language therapists trained in music-based aphasia rehabilitation techniques.	Meta-analysis demonstrated significant improvements in functional communication (mean difference [MD] = 1.45; 95% CI 0.24–2.65; p=0.02), repetition ability (MD = 6.49; 95% CI 0.97–12.00; p=0.02), and naming performance (MD = 11.44; 95% CI 1.63–21.26; p=0.02) among patients receiving music therapy compared with conventional therapy or no therapy. Findings suggested that music-supported language rehabilitation may facilitate recovery of expressive language and communication function after stroke-related aphasia. However, methodological quality among studies ranged from poor to excellent, and the authors recommended larger high-quality trials to further validate efficacy.
Wang et al. [20]	Prospective randomized clinical study involving 60 patients with first-onset ischemic stroke admitted to a rehabilitation department in Shenzhen, China between October 2017 and December 2018. Participants were randomized into a control group (n=30; 10 males, 20 females; mean age 61.02 ± 7.51 years) and a music therapy group (n=30; 8 males, 22 females; mean age 61.12 ± 7.49 years). Eligible participants had disease duration ≤3 months, established walking ability, hemiplegia with abnormal gait, and preserved cognition. Interventions were conducted in a hospital-based stroke rehabilitation setting.	Both groups received conventional stroke rehabilitation, including proprioceptive neuromuscular facilitation, neuromuscular electrical stimulation, balance training, gait training, trunk and pelvic control exercises, ankle dorsiflexion induction, and activities of daily living training for 40 minutes daily over 4 weeks. The intervention group additionally received rhythm-based music therapy three times daily for 1 hour/session. Music therapy utilized rhythmic auditory stimulation in which metronome-guided walking cadence was first established, followed by walking synchronized to familiar rhythmic music selected according to patient walking velocity. Sessions were supervised by therapists, and walkers were permitted for patients with balance difficulties. Therapy was conducted 6 days/week for 4 weeks.	Compared with controls, the music therapy group demonstrated significantly greater improvements in stride length, cadence, maximum walking velocity, gait symmetry, lower extremity motor function, balance, and treatment satisfaction after 2 weeks and at completion of therapy. At therapy completion, stride length improved to 29.36 ± 2.44 vs. 25.44 ± 1.36 (p<0.001), cadence to 71.01 ± 2.99 vs. 66.45 ± 3.01 (p<0.001), and maximum walking speed to 43.01 ± 2.54 vs. 38.14 ± 2.69 (p<0.001) in intervention versus control groups. Fugl-Meyer Assessment (FMA) lower extremity scores improved significantly (28.21 ± 4.65 vs. 24.12 ± 5.14; p=0.002), as did Berg Balance Scale (BBS) scores (42.47 ± 6.51 vs. 36.12 ± 7.14; p<0.001). Patient treatment satisfaction was also significantly higher in the music therapy group. The study concluded that rhythmic auditory stimulation through music therapy may enhance gait recovery, motor coordination, balance, and rehabilitation engagement after ischemic stroke.
Qin [21]	Randomized controlled study involving 120 hospitalized patients with ankylosing spondylitis (AS) admitted to the People’s Hospital of Anhui Province, China between March 2018 and March 2019. Participants were divided into a music intervention group (n=56; 50 males, 6 females; mean age 26.6 ± 4.5 years), a painting therapy group, and a routine treatment group. Disease duration ranged from 3 months to 16 years. Interventions were conducted in inpatient clinical and structured therapeutic environments with post-discharge follow-up.	The music intervention group received traditional Chinese music therapy in addition to standard medical treatment and counseling. Personalized music packages were developed according to traditional Chinese music categories (court music, poet music, religious music, and folk music), tailored to patient symptoms and preferences. Patients listened to music for 30 minutes every morning and before bedtime during hospitalization, with optional additional listening sessions. Music volume was maintained at 20–40 dB. Nurses introduced the cultural background and emotional context of the music and guided patients into reflective and immersive listening experiences. Weekly supportive communication sessions and telephone follow-up were conducted to reinforce adherence. The intervention course lasted 8 weeks. The painting therapy group participated in structured painting and calligraphy sessions four times weekly for 60 minutes/session in a supervised group setting.	After 8 weeks, the music therapy group demonstrated significantly greater improvements in physical and psychological functioning compared with painting therapy and routine treatment groups. Physical function scores were highest in the music group (68.82 ± 6.04 vs. 58.87 ± 6.04 vs. 45.66 ± 5.01; F=164.071, p<0.001). Significant improvements were observed in sleep and energy (F=53.979, p<0.001), physical discomfort (F=37.567, p<0.001), eating function (F=10.718, p<0.001), sexual function (F=22.155, p<0.001), and exercise/sensory function (F=75.684, p<0.001). Psychological function scores were likewise highest in the music therapy group (69.02 ± 4.62 vs. 60.98 ± 6.39 vs. 41.29 ± 7.13; F=229.535, p<0.001). Significant improvements were observed in mental tension, negative emotion, positive emotion, cognitive function, and self-esteem across groups, consistently favoring music therapy (all p<0.001). The study concluded that traditional Chinese music therapy may substantially improve both physical and psychological well-being among patients with ankylosing spondylitis and may serve as a useful adjunctive therapeutic modality.
Bains et al. [22]	Systematic review of 6 studies involving individuals with cancer and their caregivers participating in group singing interventions across community, supportive care, and cancer survivorship settings. Included studies utilized observational cohort, prospective, retrospective, randomized controlled, and crossover study designs. Detailed participant demographics and setting characteristics varied among studies and were incompletely available from the accessible abstract.	NeuroArts interventions consisted of structured group singing and choir-based activities for patients with cancer and caregivers. Singing programs were conducted in supportive group environments designed to promote psychosocial well-being and emotional support. Specific details regarding intervention duration, session frequency, session length, facilitator background, and musical repertoire were variably reported and not fully accessible from the abstract-only source.	Group singing significantly reduced anxiety levels among both individuals with cancer and caregivers. Improvements were also reported in well-being, self-efficacy, self-esteem, energy, relaxation, social connectedness, and selected health-related quality of life domains, including vitality, bodily pain, and mental health. Participants additionally demonstrated reductions in fear, anger, and confusion during longer-term follow-up compared with no-treatment groups. Effects on depression were variable, and no significant improvement in fatigue was observed. The review concluded that group singing may provide beneficial psychosocial and quality-of-life effects for patients with cancer and caregivers, although the overall methodological quality of included studies was poor.
Sihvonen et al. [23]	Narrative review of randomized controlled trials evaluating music-based neurological rehabilitation among patients with stroke, dementia, Parkinson’s disease, multiple sclerosis, and epilepsy across inpatient, outpatient, rehabilitation, nursing home, and community settings. The review synthesized 41 randomized controlled trials involving diverse neurologic populations with cognitive, motor, language, emotional, and behavioral impairments.	Music-based interventions included active and receptive modalities such as music listening, singing, dancing, rhythmic auditory stimulation (RAS), melodic intonation therapy (MIT), music-supported therapy (MST), instrument playing, improvisation, rhythmic movement therapy, tango dancing, and caregiver-implemented musical activities. Intervention duration ranged from 1 week to 1 year, depending on the neurological condition and study design. Sessions were facilitated by music therapists, music teachers, rehabilitation specialists, healthcare professionals, or caregivers. Modalities targeted motor recovery, speech rehabilitation, cognition, emotional regulation, and neuroplasticity through structured auditory-motor and emotional stimulation.	Music-based interventions demonstrated beneficial effects across multiple neurological conditions. In stroke rehabilitation, rhythmic auditory stimulation improved gait velocity (d=0.46–2.13), stride length (d=0.49–1.50), cadence (d=0.02–1.82), balance, and motor recovery, while melodic intonation therapy improved communication and object naming in aphasia. Music listening enhanced verbal memory (d=0.88), focused attention (d=0.92), mood, and neuroplasticity after stroke. In dementia, music interventions improved cognition, mood, neuropsychiatric symptoms, anxiety, depression, quality of life, and caregiver well-being. In Parkinson’s disease, tango and rhythm-based interventions improved balance, gait, mobility, and quality of life. In multiple sclerosis, rhythmic auditory stimulation and keyboard-based interventions improved gait and hand function. In epilepsy, nightly exposure to Mozart’s Sonata K.448 significantly reduced seizure frequency by 17% during treatment and 16% at one-year follow-up. The review concluded that music-based interventions represent promising adjunctive neurorehabilitation strategies capable of influencing cognition, motor performance, emotional well-being, and adaptive neuroplasticity across neurological disorders.
Teixeira-Machado & DeSantana [24]	Blinded randomized controlled clinical trial involving 27 adolescents and young adults with spastic cerebral palsy (CP), aged 15–29 years, classified as Gross Motor Function Classification System (GMFCS) levels III–V. Participants were randomized into a dance group (n=13) or control physical therapy group (n=14; analyzed n=13 after attrition). Interventions were conducted over 12 weeks with 24 total sessions (twice weekly, 60 minutes each) in rehabilitation and dance studio settings. Primary outcome measured was passive lower-limb range of motion (ROM) using a Sanny® pendular fleximeter.	The dance intervention utilized the “Técnica Aplicada Lavinia Teixeira” (TALT), integrating principles from Bartenieff, Feldenkrais, and Laban movement methods. Sessions emphasized: (1) global ROM through rhythmic floor exercises; (2) motor coordination with coordinated upper- and lower-limb movements; (3) body image and proprioception via spatial-temporal orientation activities; and (4) agility, anticipatory movement sequencing, trunk/head control, and equilibrium training. The protocol incorporated rhythmic movement, music, coordinated dance patterns, proprioceptive stimulation, and expressive motor activity designed to facilitate neuroplasticity and movement control.	The dance group demonstrated statistically significant improvements in passive ROM across nearly all hip, knee, and ankle movements compared with baseline and compared with controls. Improvements included hip flexion, extension, abduction, adduction, internal/external rotation, knee flexion/extension, and ankle dorsiflexion, plantarflexion, inversion, and eversion. The control group receiving conventional physiotherapy showed only selective ROM gains. Authors concluded that dance may positively influence musculoskeletal mobility, movement control, and functional motor capacity in CP. Proposed mechanisms included activation of motor planning networks, enhancement of neuroplasticity, integration of emotional and sensorimotor processing, improved body awareness, and increased participant engagement and psychosocial well-being. The study also emphasized improvements in self-esteem, social participation, confidence, and sense of belonging associated with dance participation.
Lundqvist et al. [25]	Multi-center randomized crossover study protocol involving adults with profound intellectual and multiple disabilities (PIMD) recruited from adult habilitation centers across four Swedish regions. Inclusion criteria included profound intellectual disability, severe sensory and motor impairment corresponding to Gross Motor Function Classification System (GMFCS) levels IV–V, age ≥18 years, and ability to participate in warm-water activities. Participants commonly exhibited spasticity, orthopedic complications, epilepsy, pain, and severe communication limitations. The intervention was conducted in warm-water rehabilitation pool environments with structured multidisciplinary supervision.	The Structured Water Dance Intervention (SWAN) combined principles of hydrotherapy, music, dance, rhythmic movement, social interaction, and passive body mobilization within a biopsychosocial rehabilitation framework. Sessions were conducted once weekly for 3 months (12 sessions total), with each session lasting approximately 40 minutes in a warm-water pool. Sessions incorporated a playlist of nine songs specifically selected to facilitate rhythmic movement, emotional engagement, relaxation, and social interaction. Passive guided dance-like body movements were performed with assistance from support persons acting as “dance partners,” supervised by physiotherapists or assistant physiotherapists trained in the SWAN protocol. Participants utilized flotation devices to optimize comfort, safety, and mobility.	The study protocol aimed to evaluate the effects of SWAN on spasticity, stress, pain, social interaction, alertness, well-being, and health-related quality of life using mixed quantitative and qualitative methods. Primary outcomes included salivary cortisol as a biomarker of stress and Modified Ashworth Scale assessments of spasticity. Secondary outcomes included assistant-rated stress and well-being scales, video analysis of social interaction and affective expression, Goal Attainment Scaling (GAS), and EuroQol five-dimension scale (EQ-5D) quality-of-life measures. The rationale for SWAN was based on prior evidence suggesting that dance and hydrotherapy may improve motor function, reduce pain and anxiety, decrease spasticity, promote relaxation, and enhance psychosocial well-being among neurologically impaired populations. Preliminary pilot findings cited by the authors demonstrated reductions in spasticity and increased alertness following SWAN participation.
Carapellotti et al. [26]	Systematic review and meta-analysis involving older adults with mild-to-moderate idiopathic Parkinson’s disease (mean Hoehn & Yahr stage 2.2; mean age 68.4 years). Included studies were conducted across diverse rehabilitation and community settings and primarily evaluated ambulatory patients with motor impairment and reduced quality of life associated with Parkinson’s disease.	Dance-based interventions included adapted tango, ballroom dance, contemporary dance, and other structured dance modalities delivered in supervised therapeutic or community programs. Interventions emphasized rhythmic movement, balance training, gait coordination, motor learning, social interaction, and self-expression. The meta-analysis incorporated nine studies evaluating different dance techniques and trial locations.	Dance interventions demonstrated improvements in motor impairment and quality of life among individuals with Parkinson’s disease. Reported benefits included gains in Unified Parkinson's Disease Rating Scale (UPDRS) motor scores, balance, gait, and postural control, with evidence suggesting potential benefit even in advanced disease stages. The review also highlighted psychosocial advantages related to social engagement and self-expression. Authors concluded that dance represents a promising adjunctive neurorehabilitative strategy for Parkinson’s disease, while emphasizing the need for larger and more diverse future studies.
Walton et al. [27]	Mixed-methods feasibility study involving 23 participants with Parkinson’s disease (PD) (mean age 70.4 years; 17 females, 6 males) recruited from Dance for Parkinson’s Disease (DfPD©) classes at Balettakademien in Stockholm, Sweden during the COVID-19 pandemic. Mean disease duration was 8 years (range 1–26 years); 57% reported good-to-very-good health and 57% had prior DfPD experience. Interventions were conducted remotely through home-based Zoom sessions as an alternative to in-person dance programs during pandemic restrictions.	Participants underwent a 10-week digital Dance for PD intervention delivered via Zoom once weekly for 60 minutes/session by a professional certified Dance for PD instructor. Sessions incorporated warm-up exercises, seated dance techniques, improvisational and choreographed movement sequences, Ballet, Flamenco, and Contemporary dance exercises, rhythmic movement training, theatrical interpretation, memorization tasks, and cool-down exercises. Dance activities emphasized rhythmic cueing, balance, motor coordination, cognition, creativity, and emotional expression. Sessions included social interaction periods before and after classes. Safety measures included continuous video monitoring, chair-assisted standing exercises, and adaptation of all movements according to participant ability and physical state.	Digital dance demonstrated high feasibility and safety, with 86% class completion and no major adverse events reported. Significant improvements were observed in quality of life measured by the Parkinson’s Disease Questionnaire-39 (PDQ-39) summary index (p<0.001), mobility subscale (p=0.016), cognition subscale (p=0.005), and depressive symptoms measured by the Hospital Anxiety and Depression Scale (HADS) depression subscale (p=0.003). Participants additionally reported subjective improvements in physical functioning (74%), overall well-being (78%), mood, balance, gait, rigidity, energy, cognition, and social connectedness. Qualitative analysis revealed themes of improved mood, “physical awakening,” cognitive engagement, emotional benefits from music, and appreciation of dance as an artistic and social activity during pandemic isolation. Limitations of digital delivery included reduced social interaction, environmental constraints, and a diminished sense of community compared with live dance.
Tao et al. [28]	Older adults with mild cognitive impairment (MCI), Alzheimer’s disease (AD), and dementia included in a systematic scoping review and meta-analysis of 29 dance intervention studies (13 randomized controlled trials) involving 1,708 participants (1,247 females, 461 males) aged 63.8 ± 5.24 to 85.8 ± 5.27 years. Studies were conducted across both clinical and community-based settings, including rehabilitation centers, outpatient programs, nursing facilities, and community elder-care environments. Clinical populations included cognitively impaired older adults with MCI, AD, and dementia-related cognitive decline.	Dance movement interventions (DMI) included structured active dance-based therapies such as aerobic dance, ballroom dance, therapeutic dance, movement improvisation, rhythmic movement training, and culturally adapted dance programs. Interventions varied across studies but commonly incorporated music-guided movement, balance training, coordination exercises, memory tasks, social interaction, and motor-cognitive integration activities. Sessions were generally supervised by dance instructors, rehabilitation specialists, therapists, or trained facilitators in group-based formats conducted over several weeks to months. The review highlighted mechanisms involving frontal lobe activation, visuospatial processing, path integration, motor planning, and entorhinal–hippocampal network engagement associated with spatial memory and executive processing.	Meta-analysis of 8 randomized controlled trials demonstrated significant improvements in global cognition, memory, balance, and depressive symptoms following dance interventions. No statistically significant improvement was observed for executive function. Dance interventions were additionally associated with enhanced mood, social interaction, quality of life, enjoyment, and psychological well-being. Authors concluded that dance represents a feasible, engaging, affordable, and non-pharmacologic complementary intervention for cognitive rehabilitation and dementia care in older adults with cognitive impairment.
Barnish and Barran [29]	Systematic review involving 56 studies (67 publications) comprising 1,531 patients with Parkinson’s disease (PD) from 12 countries, including Australia, Canada, Germany, Italy, Ireland, Japan, New Zealand, Norway, South Korea, Sweden, the United Kingdom, and the United States. Included studies involved adults with PD recruited from outpatient neurology clinics, rehabilitation programs, community-based Parkinson’s support groups, performing arts programs, and hospital-associated movement therapy settings. Sample sizes ranged from 5 to 95 participants per study (median sample size 22). Clinical populations included patients with motor symptoms, speech impairment, cognitive dysfunction, gait instability, and reduced quality of life associated with PD.	Active group-based performing arts interventions included dance (38 studies), singing interventions (12 studies), music therapy (4 studies), and theatrical interventions (2 studies). Dance modalities included tango, adapted tango, ballroom dance, PD-specific dance programs, ballet, Irish set dancing, improvisational dance, mixed-genre dance, and folk dance. Singing interventions primarily involved structured choral-based singing programs. Music therapy interventions included rhythmic musical exercises, instrumental improvisation, drumming, and combined music–movement sessions. Theatre interventions involved active theatrical performance led by professional performers. Interventions were conducted in supervised group settings by dance instructors, music therapists, professional singers, speech-language therapists, theatre performers, physiotherapists, and trained facilitators. Frequencies and durations varied considerably across studies, ranging from weekly to multiple weekly sessions over several weeks to months.	Across studies, performing arts interventions demonstrated beneficial effects on quality of life, motor function, speech, cognition, gait, balance, emotional well-being, and social participation in PD. Dance interventions showed the strongest evidence for improving motor function, balance, gait, and quality of life, particularly tango-based and PD-specific dance programs. Singing interventions improved speech intensity, phonation, vocal function, communication, and psychosocial well-being. Music therapy interventions improved motor and cognitive outcomes as well as emotional well-being, while theatrical interventions improved quality of life and emotional rehabilitation. Meta-analysis demonstrated statistically significant improvements favoring tango-based dance for UPDRS motor outcomes and Timed Up and Go (TUG) performance, as well as PD-specific dance for Parkinson’s Disease Questionnaire-39 (PDQ-39) quality-of-life scores. Authors concluded that active group-based performing arts interventions represent promising adjunctive therapeutic strategies for Parkinson’s disease, although methodological heterogeneity and lack of direct modality comparison limited definitive conclusions regarding the superiority of specific NeuroArts modalities.
Salihu et al. [30]	Quasi-experimental study involving 198 internally displaced persons (IDPs) with depressive symptoms from two IDP camps (Muna Garage and Teachers Village) in North-Eastern Nigeria. Participants were adults aged ≥18 years living in IDP camps who scored ≥10 on the depressive symptoms subscale of the Depression Anxiety Stress Scale-21 (DASS-21) and were physically capable of ambulation and participation in dance activities. The majority were female (64.1%), unemployed (93.0%), and had no formal education (74.2%). Participants experienced displacement related to ongoing ethno-religious conflict and exhibited high baseline rates of depression, anxiety, and stress.	Participants in the intervention arm received an 8-week African Circle Dance (ACD) programme combined with psychoeducation. The ACD intervention consisted of weekly 75-minute sessions incorporating group-based African traditional dance performed in a circle, accompanied by live drumming and flute music. Dance sessions included warm-up, structured Maliki traditional Kanuri dance movements involving coordinated full-body rhythmic movement, partnered dance, and cool-down phases. Sessions were facilitated by a registered dance specialist together with musicians using traditional African instruments (Ganga Kura drums and Algaita flute). Psychoeducation on stress management and coping strategies was delivered to both intervention and control groups using culturally adapted “10 Stress Busters” materials.	The intervention group demonstrated significantly greater reductions in depressive symptoms and stress compared with psychoeducation alone. Depression scores improved from severe to mild levels with a very strong group-by-time interaction effect (v = 0.33, p < 0.001), while stress demonstrated a strong interaction effect (v = 0.15, p = 0.008). Anxiety improved in both groups, but without a statistically significant additional benefit from the dance intervention. The authors proposed that the therapeutic effects were mediated through embodied movement, music-driven emotional regulation, interoception, group cohesion, stress reduction, and culturally meaningful social participation. The intervention demonstrated high feasibility and acceptability within the displaced African population, with no major adverse events reported. Authors concluded that African Circle Dance may serve as a culturally adapted complementary NeuroArts intervention for psychological rehabilitation among trauma-exposed displaced populations.
Pisu et al. [31]	Pilot randomized controlled trial involving 31 female cancer survivors and their romantic partners in the United States. Survivors were at least 3 months post-completion of primary cancer treatment, underactive (<5 days/week of exercise), and in long-term relationships. Mean survivor age was 57.9 years, with 68% diagnosed with breast cancer and 22.6% African American participants. Couples were randomized into an immediate ballroom dance intervention group or a delayed-intervention wait-list control group.	The RHYTHM (Restoring Health in You and Your Partner through Movement) intervention consisted of a 12-week ballroom dancing programme, including 10 private weekly 45-minute dance lessons and 2 practice parties conducted at a Fred Astaire Dance Studio®. Couples learned Foxtrot, Waltz, Cha-Cha, and East Coast Swing under the supervision of an experienced dance instructor. Participants were encouraged to practice dancing at home five times weekly to increase physical activity and interpersonal engagement. The intervention was theoretically grounded in the Cognitive Interaction and Intimacy Model, emphasizing trust, communication, mutuality, self-disclosure, physical touch, and shared enjoyable experiences.	Cancer survivors participating in ballroom dance demonstrated significant improvements in physical activity, mental quality of life, vitality, social functioning, and dyadic trust compared with controls. Adjusted analyses showed significant positive intervention effects for physical activity (p=0.05; effect size 0.78), mental component of quality of life (p=0.04; effect size 1.05), vitality (p=0.03; effect size 0.81), and dyadic trust (p=0.04; effect size 0.58). Survivors also reported ballroom dancing as an acceptable and enjoyable “light-intensity” activity that facilitated a gradual return to exercise after cancer treatment. Couples valued spending time together, working toward common goals, and shared emotional connection. Authors concluded that ballroom dance may serve as a feasible NeuroArts-based rehabilitation strategy capable of improving psychosocial well-being, physical activity participation, and relationship quality among cancer survivors and their partners.
Ghayomzadeh et al. [32]	Pilot pre-post feasibility study involving 10 women living with HIV (WLWH) recruited from a Positive Club in Tehran, Iran. Mean participant age was 31 ± 6 years; 40% were ≤35 years old and 60% were aged 35–50 years. Mean duration of HIV infection was 2.5 ± 1.8 years. Participants were clinically stable on antiretroviral therapy (ART) for at least 6 months and had not engaged in regular physical activity during the previous 6 months. Educational attainment included elementary (40%), middle school (30%), high school (20%), and university level (10%). The study was conducted in a sociocultural environment characterized by strong HIV-related and gender-based stigma affecting women.	Participants underwent an 8-week Aerobic Dance Training (ADT) programme performed 3 times weekly in local gyms. Sessions consisted of two high-/low-impact aerobic dance sessions and one step-board aerobic dance session weekly. Each session lasted approximately 60 minutes and included a 5–10-minute warm-up, 20–40 minutes of rhythmic aerobic dance movements, and a 5–10-minute cool-down period. Exercise intensity initially targeted 60% of age-predicted maximal heart rate and progressively increased to 80% using heart-rate monitoring via Bluetooth Polar V800 devices. Training adherence exceeded 80%. The intervention was supervised through coordination with local gym staff who monitored exercise duration, intensity, and training volume using standardized logbooks.	Aerobic dance training produced significant improvements in psychological well-being among women living with HIV. Hospital Anxiety and Depression Scale (HADS) depression scores significantly improved from 6.8 ± 3.85 to 4.25 ± 3.2 (p=0.016), while total HADS anxiety/depression scores improved from 17.3 ± 8.46 to 13.0 ± 9.04 (p=0.049). Anxiety scores demonstrated nonsignificant improvement (10.5 ± 4.77 to 8.0 ± 6.18; p=0.122). Sleep quality measures using the Pittsburgh Sleep Quality Index (PSQI) did not significantly improve overall, although sleep disturbance scores demonstrated a trend toward improvement (p=0.08). No significant changes were observed in immune function (CD4+ T-cell count: 425.23 ± 123 vs 443.61 ± 190; p=0.62), Medical Symptoms Checklist scores, or overall SF-36 quality-of-life domains. Authors concluded that aerobic dance training was feasible, safe, culturally acceptable, and potentially beneficial as a non-pharmacologic NeuroArts intervention for reducing psychological distress among women living with HIV in highly stigmatized socioreligious settings.
Davico et al. [33]	Scoping review examining the use of performing arts in suicide prevention strategies. The review screened 5,737 records and included 35 studies published between 1981 and 2021 across multiple countries, including the United States, United Kingdom, Australia, Canada, Norway, Russia, Ireland, Japan, India, and several European nations. Populations included adolescents, high school and college students, healthcare workers, school staff, gatekeepers, social workers, educators, pharmacists, medical trainees, and high-risk youth. Sample sizes ranged from 10 participants to over 33,000 participants.	Performing arts interventions included role-playing, participatory theater, dramatic improvisation, online avatar-based role-play simulations, filmed verbatim theater, storytelling, music, multimedia arts, drama, creative writing, movement-based workshops, and theater performances. Most studies used role-playing–based gatekeeper training programs such as QPR (Question, Persuade, Refer), YAM (Youth Aware of Mental Health), OSCE-style simulations, and virtual simulations with emotionally responsive avatars. Theater-based interventions included community suicide-prevention performances and interactive workshops designed to reduce stigma, increase help-seeking behavior, and facilitate emotional dialogue. Some interventions combined multiple art forms such as music, photography, visual arts, sound production, and theater.	The review found that performing arts interventions generally improved suicide-prevention awareness, self-efficacy, communication skills, help-seeking behaviors, empathy, coping skills, and gatekeeper competencies. Role-playing interventions enhanced knowledge retention and behavioral rehearsal skills, while theater-based approaches reduced stigma and facilitated emotional disclosure regarding suicide and mental illness. Several randomized controlled trials demonstrated significant benefits, particularly for role-play–enhanced gatekeeper training and the Youth Aware of Mental Health (YAM) program, which showed reductions in suicide attempts and severe suicidal ideation at 12-month follow-up. Theater interventions increased willingness to seek professional help and improved self-efficacy in discussing suicidal thoughts. Across studies, no significant adverse outcomes were reported. However, the authors emphasized that most studies were heterogeneous observational studies with limited methodological rigor, preventing definitive conclusions regarding effectiveness. The review concluded that performing arts appear feasible and promising as suicide prevention tools, but further rigorous research is needed to establish therapeutic efficacy and cost-effectiveness.
McCrary et al. [34]	Systematic review and meta-analysis including 26 studies with 779 participants (mean age 60 ± 11 years) from multiple countries, including Australia, Brazil, China, Germany, India, Italy, the Netherlands, Spain, Sweden, Thailand, Turkey, the United Kingdom, and the United States. Populations included both healthy individuals and patients with hypertension, coronary artery disease, depression, chronic kidney disease, diabetes, COPD, dementia caregiving stress, osteoarthritis, tinnitus, atrial fibrillation, breast cancer, low back pain, and heart failure. Twenty studies were randomized controlled trials and six were single-group studies.	Music-based interventions included music listening, music therapy, singing, and gospel music interventions. Music listening interventions involved recorded classical music, sedative music, meditative music, patient-selected playlists, relaxing music, and culturally tailored selections. Music therapy interventions included both active and receptive formats delivered individually or in groups. Singing interventions included community singing groups, singing for lung health, choral singing, and therapeutic singing classes. Intervention durations ranged from 3 days to 1 year, with sessions varying from daily to weekly and lasting 12 minutes to 2 hours.	Music interventions were associated with statistically significant improvements in mental health–related quality of life (HRQOL), measured using SF-36/SF-12 Mental Component Summary (MCS) scores (mean difference 2.95 points; 95% CI 1.39–4.51; p<0.001), reaching clinically meaningful thresholds. Physical HRQOL improvements were smaller but still statistically significant (PCS mean difference 1.09 points; 95% CI 0.15–2.03). Adding music interventions to treatment-as-usual further improved mental HRQOL compared with standard treatment alone (MCS mean difference 3.72 points). Benefits included improvements in mood, anxiety, depression, emotional well-being, coping, and overall quality of life across diverse clinical and healthy populations. No major publication bias or significant heterogeneity was identified. The authors concluded that music interventions provide moderate-quality evidence for clinically meaningful improvements in mental HRQOL and may serve as effective non-pharmacologic adjuncts in healthcare and public health settings.
Hauck et al. [35]	Experimental neurophysiology study involving 20 healthy right-handed adults (10 female; mean age 27.2 ± 4 years) conducted at the University Medical Center Hamburg-Eppendorf, Germany. Participants underwent painful infrared laser stimulation while cortical activity was recorded using 275-channel magnetoencephalography (MEG). The study examined neural correlates of pain modulation during different music therapy paradigms.	Two distinct music therapy approaches were evaluated: (1) Receptive music therapy, in which participants listened to personally preferred music associated with well-being and distraction from pain; and (2) Entrainment music therapy, where participants actively composed individualized “pain music” and “healing music” together with a music therapist using instruments such as piano, guitar, percussion, cello, and flutes. Participants then listened to these self-composed recordings during painful laser stimulation. Control conditions included music composed by another participant and no-sound conditions.	Music significantly modulated both subjective pain perception and pain-related cortical oscillations. Preferred music within the receptive method reduced pain-related delta-band activity in the midcingulate cortex and anterior insula, suggesting attentional distraction away from pain. In contrast, self-composed pain and healing music in the entrainment method strongly influenced gamma-band oscillations in the primary somatosensory cortex (SI). Self-composed pain music increased both pain unpleasantness ratings and gamma activity, whereas healing music reduced these effects. Source localization demonstrated that gamma oscillations were prominent in contralateral SI cortex and insula/SII regions, while delta activity localized to the cingulate cortex and insula. The findings suggest that different music therapy approaches modulate pain perception through distinct neural mechanisms: receptive music therapy primarily via distraction and attentional modulation (delta-band pathways), and entrainment therapy via active top-down modulation of early sensory pain processing (gamma-band pathways).
Hajek et al. [36]	Nationally representative cross-sectional study derived from the “Old Age in Germany (D80+)” study involving 3,181 adults aged ≥80 years in Germany, including both community-dwelling older adults and individuals residing in institutional settings such as nursing homes and care facilities. Data collection was performed from November 2020 to April 2021 using written questionnaires during the COVID-19 pandemic. Mean age was 85.6 ± 4.2 years, and 64.4% were women.	Participants were asked whether they had engaged in artistic activities during the previous 12 months, including singing, making music, painting/drawing, handwork/crafts, creative writing, photography/filming/editing, and other artistic activities. Additional details regarding the frequency and location of engagement were collected. Approximately 25.5% of participants engaged in artistic activities, most commonly singing (9.0%), making music (7.7%), painting/drawing (6.4%), creative writing (5.7%), and handwork/crafts (5.6%). Most artistic engagement occurred frequently and predominantly at home (84.8%).	Artistic engagement demonstrated significant psychosocial associations, particularly regarding loneliness and life satisfaction. Overall, artistic engagement was associated with lower loneliness among men. Singing and making music were significantly associated with lower loneliness in men, while photography/filming/editing was associated with lower loneliness among women. Artistic activities performed outside the home were associated with higher life satisfaction among the total sample and especially among women. Certain activities, such as photography/filming/editing, were associated with fewer depressive symptoms, whereas “other” artistic activities were significantly associated with higher life satisfaction. The authors concluded that artistic activities may confer psychosocial benefits in the oldest-old population through mechanisms involving emotional expression, social interaction, creativity, self-identity reinforcement, and cognitive stimulation.

**Table 15:** Visual Arts, Design, and Craft in Healthy Populations

Study	Population & Setting	NeuroArts Intervention	Outcomes
Hajek et al. [36]	Nationally representative cross-sectional study derived from the “Old Age in Germany (D80+)” study involving 3,181 adults aged ≥80 years in Germany, including both community-dwelling older adults and individuals residing in institutional settings such as nursing homes and care facilities. Data collection was performed from November 2020 to April 2021 using written questionnaires during the COVID-19 pandemic. Mean age was 85.6 ± 4.2 years, and 64.4% were women.	Participants were asked whether they had engaged in artistic activities during the previous 12 months, including singing, making music, painting/drawing, handwork/crafts, creative writing, photography/filming/editing, and other artistic activities. Additional details regarding frequency and location of engagement were collected. Approximately 25.5% of participants engaged in artistic activities, most commonly singing (9.0%), making music (7.7%), painting/drawing (6.4%), creative writing (5.7%), and handwork/crafts (5.6%). Most artistic engagement occurred frequently and predominantly at home (84.8%).	Artistic engagement demonstrated significant psychosocial associations, particularly regarding loneliness and life satisfaction. Overall artistic engagement was associated with lower loneliness among men. Singing and making music were significantly associated with lower loneliness in men, while photography/filming/editing was associated with lower loneliness among women. Artistic activities performed outside the home were associated with higher life satisfaction among the total sample and especially among women. Certain activities such as photography/filming/editing were associated with fewer depressive symptoms, whereas “other” artistic activities were significantly associated with higher life satisfaction. The authors concluded that artistic activities may confer psychosocial benefits in the oldest-old population through mechanisms involving emotional expression, social interaction, creativity, self-identity reinforcement, and cognitive stimulation.
Keyes et al. [37]	Secondary analysis of the 2019–2020 “Taking Part Survey,” a nationally representative household survey conducted in England involving 7,182 adults aged ≥16 years. Participants were recruited from community-based household settings across England prior to the COVID-19 pandemic. The sample included 3,902 females (54.3%) and 3,280 males (45.7%), spanning adolescents to adults aged ≥85 years.	Creating Arts and Crafting (CAC) activities included painting, drawing, printmaking, sculpture, photography, artistic filmmaking, computer-generated art/animation, textile crafts (e.g., embroidery, crocheting, knitting), wood crafts, pottery, calligraphy, and jewelry-making. Participants were classified as CAC-engaged if they participated in at least one artistic or craft activity within the preceding 12 months. Overall, 37.4% of participants engaged in at least one CAC activity during the prior year. Activities were community/home-based, self-directed, and not therapist-led.	Engagement in CAC significantly predicted greater subjective well-being after controlling for age, gender, deprivation index, health status, and employment. CAC participation significantly improved life satisfaction (β=0.088; ΔR²=0.0005; F=4.59; p=0.032), happiness (β=0.128; ΔR²=0.0008; F=6.71; p=0.010), and sense that life is worthwhile (β=0.218; ΔR²=0.003; F=27.5; p<0.001). CAC did not significantly predict anxiety (p=0.108) or loneliness (p=0.805). The authors emphasized that CAC engagement demonstrated wellbeing effects comparable to or exceeding some sociodemographic predictors such as employment or area deprivation, suggesting that arts and crafts may represent an accessible, low-cost public health strategy for improving population-level well-being.
Chen and Buckingham [38]	Qualitative study involving 18 older Chinese migrants living in Auckland, New Zealand (12 females, 6 males; age range 65–83 years; mean age 71 years). Participants had resided in New Zealand for 4–26 years and migrated primarily through family reunification pathways. Data were collected through in-depth interviews and photo elicitation conducted in Mandarin, Cantonese, or Hokkien between September 2022 and September 2024. Participants lived within community-based residential settings and commonly experienced postmigration stressors, including limited English proficiency, social isolation, caregiving responsibilities, and restricted social networks.	Creative engagements included gardening, painting, digital art-making, vlog/video creation, singing, knitting, carpentry, journaling, and creative writing. Gardening was the most common activity (n=10), followed by art-making (n=4), handcrafts (n=2), and creative writing (n=1). Activities were self-directed, home- or community-based, and generally not therapist-led. Participants used both traditional and digital modalities, including social media platforms, digital editing applications, online singing recordings, and smartphone-based creative production. Some activities were culturally rooted and linked to Chinese heritage, while others represented newly acquired postmigration pursuits.	Participants reported that creative engagement alleviated stress, improved emotional well-being, enhanced self-expression, fostered cultural continuity, strengthened identity, and facilitated adaptation to postmigration life. Gardening promoted emotional connection to childhood memories and enhanced sense of belonging and agency. Digital art-making and vlog creation promoted social connection and meaningful communication through online engagement. Knitting, carpentry, and painting enhanced fulfillment, purpose, cognitive engagement, and creative self-development. Creative writing and journaling facilitated emotional processing and self-exploration. Participants described creative activities as important coping mechanisms for migration-related stress, social isolation, and constrained lifestyles associated with ageing and language barriers. Authors concluded that creative engagement contributed substantially to sustaining psychological well-being and positive ageing among older migrants, while emphasizing that life-course experiences and caregiving obligations influenced participation in creative pursuits.
Gaspar da Silva M [39]	Qualitative study involving 10 immigrant women participating in a virtual group art therapy programme. Participants were immigrant women experiencing psychosocial and emotional challenges related to migration and adjustment. The intervention was conducted in an online/virtual group therapeutic setting designed to facilitate storytelling, emotional expression, social interaction, and culturally sensitive creative engagement.	An 8-session virtual art therapy programme integrating embroidery and narrative storytelling. Participants explored themes related to their past experiences, present lives, migration journeys, identity, and future aspirations through guided embroidery-based artistic creation and group storytelling. Sessions emphasized reflective narrative sharing, culturally sensitive art-making, emotional processing, and group discussion. Completed artworks were subsequently displayed in a virtual public art exhibition intended to increase public awareness regarding immigrant experiences and art therapy practices.	Participants reported that immigration experiences contributed substantially to mental health concerns and psychosocial stress. Combining embroidery-based art-making with narrative storytelling promoted psychological well-being, emotional expression, identity exploration, and meaning-making. Group-based participation fostered social reconnection, peer support, interpersonal validation, and reduction of social isolation. Embroidery was identified as a culturally sensitive therapeutic modality that facilitated communication of lived experiences and emotional processing. The virtual art exhibition additionally enhanced public understanding of immigrant experiences and expanded awareness regarding the role of art therapy practices. Findings overall supported the value of culturally responsive NeuroArts-based interventions for immigrant populations experiencing psychosocial distress.

**Table 16:** Visual Arts, Design, and Craft in Clinical Populations

Study	Population & Setting	NeuroArts Intervention	Outcomes
Caddy et al. [40]	Quantitative retrospective outcome study involving 403 psychiatric inpatients (82.4% female, 17.6% male; mean age 47.9 years) admitted to a private psychiatric hospital in Perth, Western Australia, between 2004 and 2009. Participants had attended at least six sessions of a creative activity group and no other group therapy intervention during admission. Clinical populations primarily included mood/affective disorders (64.8%), schizophrenia (14.1%), anxiety disorders (12.4%), substance use disorders, organic disorders, and personality/behavioral disorders. The average hospital stay was 19.3 days. Participants received concurrent psychiatric care, psychopharmacologic treatment, and some received electroconvulsive therapy (ECT).	Creative activity group interventions included art-, craft-, and expressive arts–based activities such as painting, beading, sewing, clay work, paper crafts, and mixed creative projects delivered in an acute inpatient psychiatric setting. Sessions were facilitated over a 5-year period by a credentialed mental health nurse with postgraduate counseling and art therapy training, together with nursing co-facilitators. Participants attended at least six sessions (~9 hours total exposure), consistent with psychotherapy dose–effect recommendations. Interventions emphasized creative self-expression, sensorimotor engagement, social interaction, emotional processing, and meaningful activity participation.	Significant improvements were observed across all psychometric outcome measures from admission to discharge. Health of the Nation Outcome Scale (HoNOS) scores improved significantly (t=24.42, p<0.001; effect size [ES]=1.26). Depression Anxiety Stress Scale-21 (DASS-21) scores significantly improved for depression (t=12.35, p<0.001; ES=0.98), anxiety (t=11.34, p<0.001; ES=0.88), and stress (t=8.52, p<0.001; ES=0.66). Quality of Life Enjoyment and Satisfaction Questionnaire (Q-LES-Q) scores improved significantly (t=12.25, p<0.001; ES=0.98), while SF-14 mental health (t=-11.39, p<0.001; ES=0.85) and vitality (t=-8.50, p<0.001; ES=0.63) scores also improved. Authors concluded that participation in creative activity groups correlated with clinically meaningful reductions in psychiatric symptom burden and improvements in quality of life, emotional well-being, and mental health functioning. Proposed mechanisms included “flow” states, distraction from rumination, non-verbal emotional expression, enhanced self-esteem, empowerment, social interaction, and activation of sensorimotor and neurocognitive pathways during creative engagement.
Kimport and Hartzell [41]	One-group pretest/posttest study involving 49 adult psychiatric inpatients from two general adult psychiatric units in a private psychiatric hospital. Participants included 28 women and 21 men aged 19–59 years (mean age 37.47 years). Ethnic composition included Caucasian/White (49%), African American/Black (22.4%), and Hispanic/Latino/Puerto Rican (18.3%) participants. Clinical populations included adults hospitalized for schizophrenia, psychotic disorders, major depressive disorder, bipolar disorder, anxiety disorders, and other severe psychiatric illnesses associated with heightened anxiety symptoms.	Participants completed a structured clay-based art therapy intervention involving creation of a clay pinch pot using either Model Magic®, Air Dry Clay, or both materials. Participants received a brief demonstration followed by up to 10 minutes of structured clay manipulation and sculpting. The intervention emphasized tactile sensorimotor engagement, structured creative expression, and active manipulation of clay materials. Participants were informed that their clay products would not be formally analyzed and could either be kept upon hospital discharge or discarded. The intervention was administered individually in a quiet meeting room within the psychiatric unit under the supervision of the study investigator/art therapist.	Significant reductions in state anxiety were observed following the clay intervention. Mean State-Trait Anxiety Inventory (STAI) state anxiety scores decreased from 46.84 pre-intervention to 39.33 post-intervention (Wilks’s lambda=0.53; F(1,47)=41.12; p=0.001). A significant gender interaction was also observed (Wilks’s lambda=0.908; F(1,47)=4.74; p=0.035), with men demonstrating greater anxiety reduction compared with women. Men had higher baseline anxiety scores (49.9) than women (44.5), though both groups reached nearly identical post-intervention scores (~39.3). Most participants (57.1%) used both clay types, and 87.8% elected to keep their completed clay artwork after discharge, suggesting meaningful attachment to the created object. The authors concluded that structured clay manipulation may serve as an effective, rapid, and feasible anxiety-reduction intervention for psychiatric inpatients. Proposed therapeutic mechanisms included emotional expression, catharsis, tactile sensorimotor regulation, increased sense of control, permanence of the clay product, and reduction of psychological distress through active creative engagement.
Gocen and Sari Ozturk [42]	Parallel-group randomized controlled trial involving 72 adolescents with type 1 diabetes mellitus (T1DM) aged 9–18 years recruited from the Pediatric Endocrinology Outpatient Clinic of Ankara Bilkent City Hospital, Türkiye, between November 2023 and August 2024. Participants were randomized into intervention (n=36) and control (n=36) groups using stratified block randomization based on age and gender. Adolescents had T1DM for at least 6 months, internet access, and no major psychiatric comorbidities or psychoactive medication use. The intervention was delivered in an outpatient/community-home hybrid setting via WhatsApp telehealth sessions.	The intervention group participated in a structured 6-week therapeutic artistic activity programme delivered individually via WhatsApp. Sessions occurred once weekly and lasted approximately 30–45 minutes. NeuroArts modalities included Zentangle drawing, mandala coloring/painting, third-person narrative communication techniques, and the “four-leaf awareness clover” mindfulness activity. Adolescents received individualized art kits containing drawing notebooks, colored pencils, graphite pencils, mandala templates, and mindfulness materials. Activities emphasized mindfulness, emotional expression, cognitive reflection, creativity, and self-awareness. The programme was conducted by a pediatric nurse with art therapy certification and supervised by a pediatric nursing academic with expertise in mindfulness and art-based psychosocial interventions.	Significant reductions in anxiety and improvements in psychological well-being were observed following the intervention. Post-intervention State Anxiety Inventory scores were significantly lower in the intervention group (32.19 ±2.75) compared with controls (45.13 ±4.84; Z=−6.669; p<0.001; Cohen’s d=3.29). Psychological Well-Being Scale scores were significantly higher in the intervention group (48.52 ±5.30) compared with controls (33.41 ±9.83; Z=−5.823; p<0.001; Cohen’s d=1.91). Within-group analyses also demonstrated significant improvement in anxiety (d=0.63) and psychological well-being (d=0.97) among intervention participants. The authors concluded that integrating therapeutic artistic activities with mindfulness and cognitive-reflective approaches provided a highly effective psychosocial intervention for adolescents with T1DM. Proposed mechanisms included stress reduction, improved self-expression, enhanced emotional regulation, mindfulness development, increased self-awareness, strengthened resilience, and improved coping with chronic illness–related psychological distress.
Ozkafacı and Eren [43]	Pretest–posttest semi-experimental study involving 8 adult female survivors of domestic physical and psychological violence diagnosed with post-traumatic stress disorder (PTSD) in Istanbul, Türkiye. Participants were referred from a private psychological counseling center and underwent 14 weekly art psychotherapy sessions between January and April 2018. Mean participant age ranged from 39 to 45 years, with all participants exposed to psychological violence and 67.5% additionally exposed to physical violence. Sessions were conducted in a group psychotherapy setting within a dedicated room designed for both artistic activity and reflective sharing.	Participants underwent a structured 14-session group art psychotherapy program centered on traditional Turkish marbling art (“Ebru”). Sessions lasted approximately 120 minutes weekly and included emotional sharing, breathing/body warm-ups, marbling creation, and group reflection. Multiple marbling modalities were used therapeutically, including stone marbling, tiger-eye marbling, nightingale nest marbling, tidal marbling, wavy marbling, mask-making with marbling, shadow marbling, tulip marbling, framed marbling, collaborative marbling, and improvisational/free marbling. Therapeutic goals included emotional expression, trauma processing, self-recognition, rebuilding self-esteem, coping with uncertainty, developing future goals, enhancing resilience, and creating symbolic representations of trauma experiences. The intervention integrated metaphorical, sensory, symbolic, and nonverbal expressive processes through color, movement, improvisation, and narrative reflection.	Significant reductions were observed in depression, anxiety, and hopelessness following the intervention. Beck Depression Scale scores improved from 25.3 ±5.16 to 9.3 ±3.14 (z=−2.041; p=0.041). Beck Anxiety Scale scores improved from 34.5 ±8.7 to 16.3 ±6.3 (z=−2.214; p=0.027). Beck Hopelessness Scale scores improved from 11.6 ±5.9 to 5.9 ±5.1 (z=−2.220; p=0.026). Qualitative analysis demonstrated that participants symbolically expressed traumatic experiences through marbling artworks, revealing themes of anger, helplessness, fear, shame, anxiety, coping, resilience, hope, self-recognition, and future planning. Over time, participants progressed from dark, chaotic imagery toward more varied colors, structured forms, future-oriented imagery, and expressions of empowerment and hope. The authors concluded that marbling art psychotherapy facilitated emotional expression, trauma externalization, social cohesion, self-awareness, coping with uncertainty, restoration of agency, and increased hopefulness in women with PTSD related to domestic violence.
Haynes [44]	Mixed-methods master’s thesis study involving 3 adult female survivors of sex trafficking aged 28–36 years in the United States. Participants had histories of severe sexual exploitation and complex trauma symptoms consistent with post-traumatic stress disorder (PTSD). The intervention was conducted in a small-group therapeutic setting over 6 weeks using trauma-informed art therapy principles. Participants were recruited from a rehabilitation/recovery environment serving women recovering from sex trafficking and sexual exploitation.	Participants underwent weekly clay-based group art therapy sessions lasting approximately 60–90 minutes over 6 consecutive weeks. Therapeutic activities used hand-building clay techniques, including coil pots, story bowls, group sculptures, patina applications, collaborative clay work, and worry stones. Sessions incorporated trauma processing, symbolic storytelling, identity exploration, emotional expression, empowerment exercises, interpersonal sharing, and collaborative art-making. The intervention emphasized tactile engagement with clay to facilitate nonverbal trauma processing, sensory integration, emotional regulation, empowerment, community-building, coping skills, and vocational/life skill development. Group psychotherapy principles such as universality, cohesion, interpersonal support, and shared recovery narratives were integrated throughout treatment.	Qualitative findings demonstrated reductions in trauma symptoms, improved emotional expression, increased self-awareness, enhanced identity formation, strengthened interpersonal trust, and the development of community cohesion among participants. Themes identified included self-thoughts, guarded boundaries, false identity/image, traumatic memories, identity reconstruction, empowerment, and community connection. Quantitative Trauma Symptom Checklist (TSC-40) data demonstrated reductions in depression, anxiety, sleep disturbance, dissociation, sexual abuse trauma symptoms, and sexual problems following intervention. Participants described clay work as grounding, emotionally expressive, calming, and empowering. Collaborative sculptural work fostered interpersonal trust, belongingness, and mutual support. Authors concluded that clay-based art therapy may represent an effective holistic NeuroArts intervention for survivors of sex trafficking by simultaneously addressing trauma recovery, emotional processing, empowerment, life skills, and social reintegration.
Nan and Ho [45]	Randomized controlled trial involving 106 adults with major depressive disorder (MDD) recruited from three Integrated Community Centres for Mental Wellness (ICCMWs) in Hong Kong. Participants were adults aged 18–60 years receiving outpatient psychiatric care and pharmacologic treatment for depression. After screening, participants were randomized into a clay art therapy (CAT) intervention group (n=53) or nondirective visual art (VA) control group (n=53). Mean age was 46.1 years in the CAT group and 44.1 years in the VA group; approximately 89% of participants were female. Most participants demonstrated moderate depressive symptoms and elevated alexithymia at baseline.	The CAT group participated in six weekly 2.5-hour clay art therapy sessions facilitated by a qualified art therapist. The intervention incorporated expressive therapies continuum principles involving kinesthetic/sensory, perceptual/affective, cognitive/symbolic, and creative processes. Participants engaged in tactile clay manipulation, sculpting, molding, symbolic creation, reflective sharing, and emotionally expressive clay-based artwork. Sessions included group discussion and reflection after art-making activities. The intervention emphasized multisensory integration, emotional regulation, somatosensory engagement, affective processing, symbolic communication, and nonverbal emotional expression. The control group underwent nondirective visual art activities, including handcraft work, mandala coloring, relaxation music, and verbal sharing facilitated by social workers.	Clay art therapy produced significantly greater improvement compared with nondirective visual art across multiple psychiatric and well-being outcomes. Significant time × group effects favored CAT for depressive symptoms measured using the Beck Depression Inventory-II Chinese version (BDI-II-C; p=0.008, partial η²=0.051), general health measured using GHQ-12 (p=0.005, partial η²=0.055), and body–mind–spirit well-being (p=0.014, partial η²=0.043). Within-group analysis demonstrated large effect size reductions in depression (Cohen’s d=−1.1 at T1; −1.2 at T2) and improvements in general health (d=−0.9 at T1; −1.1 at T2) in the CAT group. Significant improvements were also observed in positive affect, negative affect, daily functioning, tranquility, resilience, and spirituality. Alexithymia improved significantly at 3-week follow-up (p<0.001). The authors proposed that clay art therapy enhanced emotion regulation through multisensory engagement, tactile sensorimotor stimulation, affective processing, symbolic expression, cognitive-emotional integration, and autonomic nervous system regulation. The intervention was additionally associated with high adherence rates (94% completed all sessions), suggesting strong participant acceptability and feasibility in community psychiatric settings.
Yazici et al. [46]	Randomized controlled single-blind study involving 60 chronic stroke patients undergoing inpatient physical therapy and rehabilitation in a Training and Research Hospital in Türkiye between August and September 2022. Participants were randomized into an experimental group (n=30) and control group (n=30). Mean age was 66.8 ± 9.96 years in the experimental group and 61.2 ± 13.81 years in controls. Participants had chronic cerebrovascular disease with stroke duration >6 months and unilateral hemiplegia. Patients with severe visual, hearing, speech, perception, or psychiatric disorders were excluded. Most participants were unemployed, living with family, and had additional chronic illnesses such as hypertension or diabetes. The intervention was conducted within a structured inpatient rehabilitation setting where all patients simultaneously received routine physical therapy and rehabilitation.	The experimental group received clay art therapy in addition to standard physical therapy. Clay therapy was administered twice weekly for 8 weeks, with 60-minute sessions integrated into rehabilitation care. The intervention combined Rogers’ person-centered theory and Rice & Greenberg’s process-experiential approach using an ADDIE framework (Analyze, Design, Develop, Implement, Evaluate). Sessions were facilitated by a researcher trained and certified in clay art therapy, together with psychiatric nursing staff trained in art therapy. Activities progressed through structured therapeutic stages involving tactile exploration, free clay manipulation, shaping desired objects, ornament creation, painting, beading, and symbolic artistic completion. Participants engaged in squeezing, rolling, pulling, shaking, molding, and painting clay objects using repetitive sensorimotor and expressive artistic movements. Sessions additionally included unstructured interviews regarding emotional reactions, daily activities, and psychological experiences. The control group received only routine physical therapy and rehabilitation 5 days weekly for 40 sessions total without art therapy exposure.	Clay therapy significantly reduced depression and hopelessness compared with physical therapy alone. Post-intervention Beck Depression Inventory scores were significantly lower in the experimental group (9.067 ±5.362) compared with controls (25.033 ±5.499) (t(58)=−11.386; p<0.001). Beck Hopelessness Scale scores were also significantly lower in the experimental group (8.000 ±2.421) versus controls (15.000 ±2.853) (t(58)=−10.247; p<0.001). Significant improvements were additionally observed in hopelessness subdomains, including feelings about the future (0.967 vs 2.967; t=−8.704; p<0.001), loss of motivation (2.667 vs 6.400; t=−12.322; p<0.001), and future expectations (3.567 vs 4.667; t=−5.562; p<0.001). The authors proposed that clay therapy improved emotional regulation, sensorimotor engagement, self-expression, self-confidence, motivation, future orientation, and psychological adaptation in chronic stroke patients. The tactile and repetitive nature of clay manipulation was suggested to enhance both emotional processing and rehabilitation-related motor engagement while promoting hopefulness and psychosocial recovery.
Zhao et al. [47]	Cluster-randomized controlled clinical trial involving 93 older adults with mild cognitive impairment (MCI) aged ≥60 years recruited from the Departments of Geriatrics and Neurology at Fujian Provincial Hospital, China, between January 2015 and March 2016. Participants complained of memory impairment or suspected cognitive decline and met DSM-IV–based criteria for MCI. Subjects were randomized into a Creative Expression (CrExp) therapy group (n=48) or a control group receiving standard cognitive rehabilitation (n=45). Mean age was approximately 69–71 years, and participants were followed for 16 weeks with additional 6-month follow-up assessments.	The CrExp intervention consisted of 25 group sessions delivered over 16 weeks by professional therapists. Sessions included: (1) 5–10 minute interactive warm-up activities, (2) 10-minute drawing tasks, (3) 30-minute core storytelling-based creative expression therapy, and (4) reflective discussion/conclusion. The CrExp programme integrated storytelling, drawing, symbolic imagery, visual-spatial processing, imagination, emotional narration, and artistic expression using a structured 5-step narrative framework involving picture interpretation, scene description, story development, characterization, and story endings. Participants additionally used symbols, colors, lines, graphs, and drawings to express emotions and construct narratives. The intervention was grounded in dual-coding theory, emphasizing simultaneous verbal and visual cognitive processing. The control group underwent standard cognitive rehabilitation involving finger exercises, cognitive strategy training, games, and educational activities over an equivalent number of sessions.	The CrExp group demonstrated significantly greater improvement compared with controls across multiple cognitive domains at postintervention and sustained benefits at 6-month follow-up. Significant postintervention improvements favored CrExp in global cognitive functioning measured by Montreal Cognitive Assessment (MoCA; F=21.47; p<0.001), memory using Chinese Version Auditory Verbal Learning Test (CVAVLT immediate recall F=4.81, p=0.023; delayed recall F=3.98, p=0.012), language/verbal fluency (CVCVFT; F=3.91, p=0.017), executive functioning using Digit Span Test (DST; F=23.35, p<0.001), attention/executive processing using Trail Making Test-B (TMT-B; F=3.29, p=0.030), activities of daily living (CVADL; F=2.78, p=0.041), and memory satisfaction (MSQ; F=18.39, p<0.001). At 6-month follow-up, sustained improvements remained significant for global cognition, delayed recall memory, executive functioning, attention, activities of daily living, and memory satisfaction. Authors concluded that storytelling- and drawing-based creative expression therapy may enhance neuroplasticity, communication, executive function, working memory, visual imagery processing, emotional engagement, and social interaction in older adults at risk of dementia. The intervention was proposed as a potentially cost-effective adjunctive nonpharmacologic NeuroArts strategy for MCI populations.
Rice et al. [48]	Mixed-methods pre–post environmental intervention study conducted in an urban general practice in Bristol, United Kingdom, involving approximately 9,200 registered patients. Patient surveys included 1,118 respondents before relocation and 954 respondents after relocation to a new purpose-built facility. Participants included adult patients attending GP consultations and multidisciplinary healthcare staff, including physicians, nurses, administrative staff, receptionists, and managers. The original setting was a converted Victorian house characterized by cramped consultation rooms, poor privacy, excessive noise, limited space, and inadequate décor. The intervention setting was a purpose-built enhanced primary care facility emphasizing spaciousness, lighting, acoustics, furnishings, privacy, comfort, and commissioned artwork integrated into the architectural environment.	The NeuroArts/environmental intervention consisted of relocation into a newly designed primary care facility incorporating architectural aesthetics, healing environmental design, visual arts, calming acoustics, natural lighting, decorative elements, and commissioned artwork. The enhanced environment included larger consulting rooms (up to 22 m² versus 14.8 m² previously), quieter waiting spaces, increased privacy, comfortable furnishings, calming décor, background music, plants, informal seating, domestic-style room design, and a large aquarium as an integrated artistic installation. An art consultant collaborated with clinicians to commission multiple site-specific artworks funded by Arts Council England and related arts organizations. The intervention was continuous and embedded within the healthcare environment rather than delivered in discrete sessions. Patient and staff responses were assessed using questionnaires, interviews, and focus groups before and after relocation.	Relocation to the enhanced NeuroArts-informed healthcare environment was associated with significant improvements in patient anxiety, satisfaction, staff workplace satisfaction, and perceived doctor–patient communication. Spielberger State-Trait Anxiety Inventory (STAI) scores significantly improved both before consultation (mean difference 0.72; 95% CI 0.37–1.08; p<0.001) and after consultation (mean difference 0.37; 95% CI 0.03–0.72; p=0.033). Satisfaction with reception/waiting areas improved significantly (mean difference 6.46; 95% CI 5.97–6.95; p<0.001), while consultation room satisfaction also increased markedly (mean difference 3.80; 95% CI 3.44–4.15; p<0.001). Patient ratings of doctor–patient communication improved significantly (mean difference 0.88; 95% CI 0.30–1.46; p=0.003). Staff workplace satisfaction increased substantially after relocation and remained elevated at 11-month follow-up, particularly among healthcare professionals with personalized workspaces. Qualitative findings identified greater space, increased natural light, calming ambience, privacy, artwork, aquarium installations, modern appearance, reduced noise, and more homelike consulting rooms as major contributors to reduced stress and improved emotional comfort. Authors concluded that NeuroArts-informed healthcare architecture and environmental aesthetics may positively influence emotional regulation, stress reduction, therapeutic communication, patient confidence, professional identity, workplace well-being, and overall healthcare experience in primary care environments.

**Table 17:** Literature-Based Interventions in Healthy Populations ADHD: Attention-Deficit/Hyperactivity Disorder

Study	Population & Setting	NeuroArts Intervention	Outcomes
Zhang et al. [49]	Randomized controlled study involving 132 adults recruited to examine the mental health effects of expressive writing interventions; 100 participants (76%) completed the study protocol, including control (n=31), replay expressive writing (n=34), and rehearsal expressive writing (n=35) groups. Participants were community-based adults assessed at baseline, after completion of the intervention, and at 1-month follow-up. The study evaluated psychological adjustment, emotional symptoms, coping behaviors, and social functioning within a non-clinical community/experimental research setting.	Participants underwent 4 consecutive days of structured expressive writing interventions lasting 15 minutes daily. The rehearsal expressive writing condition involved writing about future imagined interpersonal interactions and mentally rehearsing anticipated conversations or events. The replay expressive writing condition involved reflective writing about prior interpersonal experiences and past interactions. The control group completed neutral writing tasks. Interventions were self-administered within a structured experimental framework emphasizing emotional expression, cognitive processing, narrative construction, anticipatory simulation, and reflective written storytelling. Writing expressiveness was measured longitudinally throughout the study.	Significant improvements over time were observed for psychological adjustment measured using the Mental Health Inventory-18 (MHI-18), self-perceived stress, PROMIS depression scores, PROMIS anxiety scores, and self-blame coping measures. Rehearsal expressive writing demonstrated differential coping effects compared with replay writing, including decreased behavioral disengagement and increased emotional venting. Participants in the rehearsal group consistently demonstrated significantly higher perceived writing expressiveness compared with the other groups throughout follow-up. Findings suggested that anticipatory narrative rehearsal through expressive writing may enhance emotional processing, coping flexibility, and psychological adaptation. Authors concluded that rehearsal expressive writing represents a promising, low-cost, accessible NeuroArts-based intervention capable of improving emotional well-being and adaptive coping through structured narrative imagination and written self-expression.
Sun et al. [50]	Large-scale longitudinal cohort study using data from the Adolescent Brain Cognitive Development (ABCD) Study involving 10,243 young adolescents aged approximately 9–13 years recruited across 21 research sites in the United States. Participants underwent comprehensive cognitive, behavioral, psychiatric, neuroimaging, and genetic assessments within a population-based developmental neuroscience framework. The study evaluated early childhood reading-for-pleasure (RfP) experiences, neurocognitive performance, psychopathology scores, brain structural measures, and longitudinal developmental outcomes.	NeuroArts-related literacy intervention conceptualized as early-initiated childhood reading for pleasure (RfP). Participants’ caregivers reported the duration of children’s recreational reading exposure (“For how many years has your child read for pleasure?”) and average weekly reading duration (“Approximately how many hours per week does your child spend reading for pleasure?”). The intervention domain included sustained voluntary reading exposure during childhood involving cognitively enriching literary engagement, language acquisition, narrative processing, visual-symbolic integration, and independent leisure reading habits. Longitudinal analyses examined cumulative early reading exposure, weekly reading duration, neurodevelopmental correlates, structural MRI findings, twin analyses, and Mendelian randomization analyses.	Early childhood reading for pleasure was significantly associated with superior neurocognitive and mental health outcomes in adolescence. Higher early RfP correlated with improved crystallized cognition, total cognition, fluid cognition, verbal learning, speech development, school performance, and academic grades, while also correlating with lower psychopathology scores, including attention problems, ADHD symptoms, conduct problems, externalizing behaviors, aggression, depressive symptoms, stress symptoms, and total behavioral problems. Early RfP was additionally associated with reduced screen time and improved sleep duration. Neuroimaging demonstrated moderately increased total brain volume, intracranial volume, cortical area, cortical volume, and enlargement of language- and cognition-related regions, including the temporal cortex, supramarginal gyrus, angular gyrus, frontal regions, anterior cingulate cortex, insula, thalamus, ventral diencephalon, and putamen. Longitudinal analyses showed that higher baseline early RfP predicted improved crystallized cognition and reduced attention problems two years later. Mediation analyses demonstrated that identified cortical and subcortical structures significantly mediated associations between early RfP and improved cognition and psychopathology outcomes. Approximately 12 hours/week of reading was identified as cognitively optimal. Mendelian randomization analyses suggested beneficial causal associations between early RfP and adult cognitive performance and left superior temporal cortical structure.

**Table 18:** Literature-Based Interventions in Clinical Populations

Study	Population & Setting	NeuroArts Intervention	Outcomes
Wu et al. [51]	Randomized controlled trial involving 112 breast cancer patients undergoing chemotherapy. Participants were randomly assigned to either a standard expressive writing group (n=56) or a prolonged expressive writing group (n=56). The study was conducted in an oncology/chemotherapy treatment setting and evaluated longitudinal physical, psychological, and quality-of-life outcomes among patients experiencing cancer-related emotional stress during active treatment.	NeuroArts intervention utilizing structured expressive writing therapy focused on emotional disclosure and narrative processing of cancer-related distress. The standard expressive writing group followed the classic Pennebaker paradigm, writing for at least 20 minutes daily across 4 consecutive days (4 sessions total). The prolonged expressive writing group completed a higher-dose modified intervention consisting of writing for at least 20 minutes, 3 times weekly over 4 weeks (12 sessions total), with flexibility regarding consecutive or non-consecutive writing days. Participants in both groups were instructed to write about emotionally upsetting, stressful, or traumatic experiences related to breast cancer, including emotional reactions, fears, and psychological responses to illness and treatment. Outcomes were assessed longitudinally at baseline and at 1-month, 3-month, and 6-month follow-up intervals.	No statistically significant differences were identified between the standard expressive writing group and the prolonged expressive writing group regarding quality of life, physical well-being, or psychological well-being at any measured follow-up point (all p > 0.05). Both groups demonstrated significant declines in quality of life over time during chemotherapy treatment (F = 40.64, p < 0.001), suggesting that increasing the “dose” or frequency of expressive writing did not confer additional measurable benefit compared with the shorter standard expressive writing protocol. Findings suggested that prolonged expressive writing exposure may not enhance therapeutic outcomes beyond those achieved through conventional expressive writing interventions among breast cancer patients receiving chemotherapy. The study nevertheless reinforced expressive writing as a feasible, low-cost psychosocial NeuroArts intervention capable of facilitating emotional disclosure and reflective coping during cancer treatment.
Nesterova et al. [52]	Single-institution phase II randomized controlled study involving 60 adult patients with cancer of any type or stage, of whom 50 were evaluable for analysis. Participants were randomized in a 2:1 ratio to a Creative Writing Workshop (CWW) intervention group (n=34 evaluable) or an active control group (AC; n=17 evaluable). The study was conducted in an oncology setting over a total duration of 6 months, including an 8-week intervention period and up to 3 months of follow-up. Participants were adult oncology patients experiencing psychosocial and emotional distress associated with cancer diagnosis and treatment.	NeuroArts intervention consisted of a structured group-led Creative Writing Workshop (CWW) delivered across 4 sessions conducted bimonthly over 8 weeks. Sessions were facilitated in a group format emphasizing reflective writing, emotional disclosure, narrative construction, imaginative expression, and interpersonal sharing. Participants engaged in guided creative writing exercises designed to explore emotional experiences related to cancer, identity, coping, meaning-making, and psychological adjustment. The active control group independently completed writing exercises at home using a writing guidebook over an equivalent schedule of four sessions across 8 weeks without group facilitation or interactive therapeutic discussion. Mood and psychological outcomes were assessed longitudinally using the Emotional Thermometer Scale (ETS), Patient Health Questionnaire-9 (PHQ-9), and Generalized Anxiety Disorder-7 (GAD-7).	Patients participating in the Creative Writing Workshop demonstrated significant immediate improvement in overall mood as measured by the Emotional Thermometer Scale (ETS) following sessions (postclass vs preclass scores: p<0.0001; 95% CI −4.31 to −2.47). Significant improvements were observed across multiple ETS emotional subdomains, including distress (p=0.0346; 95% CI −2.6 to −0.1), anxiety (p=0.0366; 95% CI −4.1 to −0.2), depression (p=0.0441; 95% CI −3.9 to −0.1), and anger (p=0.0494; 95% CI −3.3 to 0). No significant improvements were observed in the active control group. No statistically significant changes were identified in PHQ-9 or GAD-7 scores between groups over the study period. Findings suggested that group-based creative writing interventions may provide short-term emotional and mood-related benefits for oncology patients through narrative expression, emotional processing, interpersonal support, and reflective meaning-making, even when broader depressive and anxiety symptom scales remain unchanged.
Wright et al. [53]	Multisite pragmatic cluster randomized controlled trial involving 249 autistic children aged 4–11 years recruited from 87 schools across Yorkshire and the Humber, United Kingdom. Participants included 129 children allocated to the Social Stories™ intervention group and 120 children receiving care as usual. Recruitment additionally involved caregivers, teachers, educational professionals, NHS trusts, autism support groups, and community publicity. The intervention was implemented within mainstream and special educational school settings during the COVID-19 pandemic. Participants were children with a formal diagnosis of autism spectrum disorder experiencing social communication and behavioral challenges requiring individualized social support.	NeuroArts-related intervention utilized Carol Gray’s Social Stories™, a highly personalized narrative-based psychosocial intervention designed to improve social understanding, communication, emotional regulation, and behavioral adaptation in autistic children. Educational professionals and caregivers underwent structured training involving psychoeducation and implementation strategies for Social Stories. Individualized stories were specifically written according to contextualized social goals and the child’s unique social information needs. Stories incorporated narrative structure, visual/verbal social cues, behavioral modeling, emotional interpretation, and guided perspective-taking. Interventionists read the Social Story™ with the child at least six times over a 4-week period within the school setting. The intervention emphasized narrative processing, repeated guided exposure, emotional contextualization, and individualized social cognition support.	At 6 months, the intervention demonstrated a small nonsignificant improvement in teacher-reported Social Responsiveness Scale-2 (SRS-2) T-scores compared with care as usual (estimated difference −1.61; 95% CI −4.18 to 0.96; p=0.220), slightly favoring the Social Stories group. Parent-reported secondary outcomes generally showed minimal differences between groups, with no clinically meaningful improvements in anxiety, depression, social responsiveness, general health, or parental stress. However, children receiving Social Stories met individualized behavioral and social goals significantly more frequently than controls (difference 0.97; 95% CI 0.21–1.73; p=0.012). The intervention was additionally associated with potential cost savings (−£191 per child; 95% CI −767.7 to 337.7) while maintaining comparable quality-adjusted life-years. Economic analyses suggested a 75% probability of Social Stories being cost-effective at a willingness-to-pay threshold of £20,000 per quality-adjusted life-year gained. Authors concluded that although Social Stories did not significantly improve broad social responsiveness or emotional health outcomes, they may serve as a low-cost, individualized NeuroArts-informed narrative intervention useful for addressing specific behavioral goals and facilitating communication between autistic children and educational staff.
Zhu et al. [54]	Prospective pilot randomized feasibility study involving 16 adult patients with metastatic or unresectable cancer. Participants were randomized in a 2:1 ratio to either an intervention arm (IA; n=11) receiving Creative Writing Workshops (CWW) or a standard-of-care (SOC) control group (n=5). The study was conducted within an oncology setting over a 4-week intervention period. Participants were patients with advanced-stage cancer experiencing substantial psychosocial stressors related to progressive malignancy and ongoing treatment.	NeuroArts intervention consisted of structured Creative Writing Workshops (CWW) delivered weekly over 4 weeks. Participants in the intervention arm attended four 2-hour group-based creative writing sessions facilitated within a therapeutic workshop environment. Sessions emphasized reflective writing, emotional disclosure, narrative expression, storytelling, interpersonal discussion, imaginative processing, and meaning-making related to cancer experiences and psychological distress. Participants in the SOC group did not receive writing intervention exposure. Emotional outcomes were assessed using the validated Emotional Thermometer Scale (ETS) before and after intervention sessions. Feasibility outcomes included attendance and adherence rates.	The intervention demonstrated feasibility among patients with advanced cancer, with 63% of participants in the intervention arm attending at least 75% of workshop sessions. Patients undergoing Creative Writing Workshops demonstrated a trend toward overall mood improvement compared with standard-of-care controls when comparing initial and final Emotional Thermometer Scale (ETS) scores. Within the intervention group, significantly lower postclass total ETS scores were observed relative to preclass scores, indicating immediate mood improvement following workshop participation. Additionally, a significant decreasing trend in preclass ETS scores over time suggested progressive emotional benefit across repeated sessions. Findings supported the feasibility and potential psychosocial value of group-based creative writing interventions for patients with advanced cancer, particularly through mechanisms involving emotional expression, narrative processing, reflective coping, interpersonal support, and meaning-centered therapeutic engagement.
Segar et al. [55]	Case series involving military veterans receiving palliative care services in the United States. Participants were adult veterans with serious or life-limiting illnesses managed within palliative care settings, including individuals with advanced chronic disease, psychosocial distress, existential suffering, and end-of-life concerns. The intervention was conducted within interdisciplinary palliative care and supportive care environments emphasizing holistic psychosocial and spiritual care.	NeuroArts intervention involved individualized poetry-based therapeutic encounters integrated into palliative care. Poetry interventions included reading, discussing, selecting, and creating poems tailored to the veterans’ emotional experiences, personal histories, military identity, suffering, grief, spirituality, meaning-making, and end-of-life reflections. Sessions were facilitated within clinical palliative care interactions by healthcare professionals incorporating narrative medicine and expressive humanities approaches. Poetry served as a medium for emotional expression, reflective storytelling, existential exploration, interpersonal communication, and therapeutic dialogue between patients, clinicians, and caregivers.	Poetry interventions were associated with reduced emotional distress, enhanced emotional expression, improved communication between patients and healthcare providers, strengthened interpersonal connection, and facilitation of meaning-making during serious illness and end-of-life care. Veterans used poetry to articulate difficult emotions, traumatic memories, fears, hopes, identity-related experiences, and existential concerns that were otherwise difficult to verbalize directly. Poetry additionally supported dignity preservation, reflective coping, psychosocial comfort, and therapeutic rapport within palliative care encounters. Authors concluded that poetry-based NeuroArts interventions may serve as valuable adjunctive tools in palliative medicine by facilitating narrative expression, emotional processing, empathic communication, and holistic patient-centered care among veterans with advanced illness.
Goddu et al. [56]	Qualitative study involving African-American adults with diabetes mellitus participating in the Diabetes Empowerment Program (DEP) on the South Side of Chicago, USA. The DEP was implemented through six partner clinics serving predominantly working-class African-American communities experiencing major diabetes disparities. Participants attended weekly small-group diabetes empowerment classes for 10 weeks, followed by monthly support meetings. The majority of participants were women. The intervention was situated within a culturally tailored community diabetes self-management and shared decision-making programme.	Narrative-based NeuroArts intervention integrated three modalities within the DEP: film-based storytelling, personal storytelling, and role-play. The programme incorporated an 11-minute culturally tailored film featuring African-American actors and narratives related to diabetes self-management and shared decision-making. Personal storytelling reflected the African-American oral tradition of “testifying,” allowing participants to share lived experiences and coping narratives related to diabetes. Role-play scenarios simulated challenging diabetes-management and healthcare communication situations to build practical behavioral skills. Narrative interventions emphasized cultural embeddedness, emotional engagement, identification with characters, transportation into stories, social discussion, rehearsal of messages, and reciprocal peer support.	Narrative interventions were perceived to improve self-efficacy, knowledge acquisition, practical problem-solving skills, attitudes toward diabetes management, confidence, social support, and behavioral motivation. Participants reported that culturally embedded storytelling improved retention of information, facilitated sharing of practical management strategies, and enhanced tangible skills for negotiating challenging situations through role-play and peer modeling. Social proliferation — discussion and reinforcement of stories within the group — emerged as a particularly influential mechanism promoting behavior change, empowerment, confidence, perceived social norms, and reciprocal support. The authors proposed a conceptual model whereby narrative interventions influence behavior through transportation, identification, and social proliferation mechanisms, ultimately facilitating diabetes self-management and empowerment in African-American communities.
Dingle et al. [57]	Quasi-experimental study involving 39 adults with chronic mental health conditions recruited from community mental health support organizations in Australia. Participants included adults with long-standing psychiatric disorders, including depression, anxiety disorders, bipolar disorder, schizophrenia-spectrum disorders, and other chronic mental health conditions receiving ongoing psychosocial support services. Participants engaged in structured arts-based group programmes conducted within community and group rehabilitation settings.	Participants engaged in two multimodal NeuroArts interventions: choir singing and creative writing group programmes. Activities were delivered in structured community-based group formats emphasizing emotional expression, creativity, interpersonal interaction, reflective processing, and shared artistic participation. Choir singing involved collaborative vocal performance, musical engagement, breathing synchronization, emotional expression through song, and social connectedness. Creative writing activities involved narrative expression, reflective writing, storytelling, emotional disclosure, and imaginative literary engagement. Interventions emphasized participation rather than artistic skill, with repeated sessions conducted longitudinally to examine emotional regulation and affective change over time.	Arts-based participation was associated with significant increases in positive emotions and reductions in negative emotions among adults with chronic mental health conditions. Participants demonstrated sustained reductions in negative affect and improvements in emotion regulation capacities following engagement in choir singing and creative writing programmes. Findings suggested that multimodal artistic engagement promoted emotional expression, social bonding, interpersonal connectedness, self-reflection, psychological coping, and affective regulation. The group-based nature of the interventions additionally fostered social identity formation, peer support, belongingness, and shared emotional experiences. The authors concluded that choir singing and creative writing may represent effective community-based NeuroArts interventions supporting emotional well-being and psychosocial recovery in individuals with chronic psychiatric illness.
Zachariae and O’Toole [58]	Systematic review and meta-analysis conducted in Denmark evaluating randomized controlled trials of expressive writing interventions among adult cancer patients and cancer survivors. Included studies involved heterogeneous oncology populations across multiple cancer types, treatment stages, and survivorship settings. The review synthesized findings from controlled expressive writing intervention studies conducted in clinical and survivorship contexts. The study followed PRISMA recommendations and systematically searched PubMed and PsycINFO databases.	NeuroArts intervention consisted of expressive writing interventions (EWI) based primarily on the Pennebaker paradigm. Across included studies, participants typically wrote for 15–20 minutes across several sessions regarding emotionally significant, stressful, or traumatic experiences related to cancer diagnosis, treatment, emotional distress, fears, coping, and illness-related experiences. Interventions emphasized emotional disclosure, written emotional expression, cognitive processing, narrative construction, and reflective meaning-making. Comparator groups generally involved neutral or non-emotional writing tasks. The meta-analysis evaluated both psychological and physical health outcomes as well as moderating contextual factors such as social support and emotional constraints.	Meta-analysis demonstrated no overall statistically significant effects of expressive writing interventions on psychological outcomes, physical health outcomes, or quality-of-life measures among cancer patients and survivors. Most included studies reported null findings, although isolated studies suggested possible improvements in pain, sleep, and general physical or psychological symptoms. The authors identified potential moderator effects, indicating that efficacy may depend on contextual and interpersonal factors such as levels of social support or social constraints. Findings suggested that expressive writing interventions are generally feasible, safe, accessible, and inexpensive for oncology populations, but broad clinically meaningful benefits were not consistently demonstrated across unselected cancer populations. Authors concluded that expressive writing may still offer therapeutic value for selected subgroups of patients, particularly those with lower emotional support or greater emotional inhibition, while emphasizing substantial heterogeneity across study designs, patient populations, and intervention protocols.
King et al. [59]	Qualitative pilot feasibility study involving 11 individuals participating in psychosocial rehabilitation programmes provided by Richmond Fellowship Queensland in Brisbane, Australia. Participants were adults with severe mental illness, predominantly middle-aged and female, with clinician-reported diagnoses including schizophreniform disorders, major mood disorders, and personality disorders. Participants were enrolled in rehabilitation services designed for individuals with moderate-to-severe psychosocial impairment related to chronic psychiatric illness.	NeuroArts intervention consisted of a structured creative writing workshop developed collaboratively by a professional writer and a mental health academic. The intervention included three sessions conducted over three weeks, with each session lasting at least two hours. Workshops emphasized the writing process and technique rather than symptom-focused disclosure. Participants engaged in “life writing,” autobiographical storytelling, reflective narrative construction, descriptive writing, sequencing, and creative literary exercises. The programme incorporated didactic instruction regarding sentence structure, descriptive language, and writing craft, alongside interactive peer discussion, feedback, and group sharing of written work. Participants were encouraged to focus on positive or recovery-oriented themes and were provided with writing kits and ongoing written feedback from the facilitator between sessions.	Participants reported that creative writing promoted emotional expression, recovery-oriented identity formation, self-esteem, agency, reflective processing, concentration, and cognitive engagement. Narrative construction was theorized to facilitate recovery by helping participants re-author personal narratives and regain a sense of self as “protagonist” rather than being dominated by illness identity. Authors proposed that writing promoted coherence, predictability, control, and development of subjectively workable narratives (“bricolage”) through structured narrative construction and imaginative processing. Additional perceived benefits included enhanced confidence, social connection, interpersonal validation, cognitive remediation, concentration, and psychosocial rehabilitation. Participants valued the involvement of a professional writer rather than a purely clinical facilitator, as this reinforced an identity as a “writer” rather than as a psychiatric patient. Authors concluded that creative writing may represent an underutilized, low-cost NeuroArts intervention capable of supporting recovery, cognitive engagement, identity reconstruction, and psychosocial rehabilitation in severe mental illness.
De Vecchi et al. [60]	Scoping review examining digital storytelling interventions across diverse mental health populations and settings in multiple countries, including Australia, Canada, New Zealand, the United Kingdom, the United States, and Zimbabwe. Included populations encompassed individuals with psychosis, severe mental illness, dementia, trauma-related disorders, Indigenous communities, refugee women, adolescents, individuals with HIV, substance-use–related behaviors, and psychosocial disability. Studies were conducted in community, educational, rehabilitation, mental health service, participatory research, and digital/online environments.	NeuroArts intervention consisted of digital storytelling, a multimodal narrative arts approach integrating personal storytelling with digital media, including photographs, voice recordings, video, music, narration, and visual imagery. Participants created autobiographical digital narratives centered on lived experiences, trauma, resilience, identity, psychosis, cultural experiences, coping, recovery, and mental health challenges. Interventions frequently incorporated collaborative workshops, participatory-action methodologies, peer sharing, reflective discussion, multimedia editing, and public dissemination of digital stories. Specific modalities included oral storytelling, visual storytelling, digital media production, culturally embedded narratives, and reflective autobiographical expression.	Across studies, digital storytelling interventions were associated with improvements in emotional expression, self-reflection, empowerment, resilience, identity formation, social connectedness, cultural preservation, recovery orientation, coping skills, and psychological well-being. Participants commonly described the process as therapeutic, healing, transformative, empowering, and conducive to emotional release and meaning-making. Digital storytelling additionally promoted interpersonal dialogue, peer support, intergenerational connection, stigma reduction, advocacy, communication with healthcare professionals, and increased understanding of lived experiences among educators and clinicians. Several studies highlighted enhanced cultural identity and resilience among Indigenous youth and communities, while others demonstrated improved reflection on stress, trauma, substance use, HIV-related experiences, and psychosis. The review concluded that digital storytelling represents a flexible and culturally adaptable NeuroArts modality capable of supporting mental health recovery, emotional processing, empowerment, education, and social inclusion through multimodal narrative expression and participatory creative engagement.

**Table 19:** Cultural Interventions in Healthy Populations

Study	Population & Setting	NeuroArts Intervention	Outcomes
Binnie [61]	An observational mixed-methods study conducted in museum and art gallery settings in the United Kingdom involving museum visitors, frequent museum attendees, infrequent visitors, and museum staff. The study included two separate investigations examining anxiety and well-being responses associated with viewing art within museum environments. Participants underwent repeated anxiety assessments and qualitative interviews exploring perceptions of well-being, emotional responses, and museum experiences.	NeuroArts intervention consisted of passive visual arts exposure within museum and gallery environments. Participants viewed artworks and exhibitions within museum settings and subsequently completed psychological assessments and interviews. The intervention incorporated both aesthetic engagement with visual art and immersion within the broader museum environment, including architectural space, exhibition design, interpretation, and environmental ambience. Anxiety levels were evaluated using the State Anxiety Inventory (SAI) and Trait Anxiety Inventory (TAI) before and after art viewing experiences. Qualitative interviews additionally explored subjective experiences of relaxation, meaning-making, emotional responses, and wellbeing associated with museum attendance.	Statistical analyses demonstrated significantly lower post-viewing State Anxiety Inventory (SAI) scores compared with Trait Anxiety Inventory (TAI) scores, suggesting that participants experienced lower anxiety after viewing artworks within museum environments than they typically experienced in everyday life (p<0.05). Frequent museum visitors demonstrated significant reductions in anxiety before versus after viewing artwork, whereas infrequent visitors showed less consistent changes. Qualitative findings indicated that participants commonly described museum environments and art viewing as relaxing, calming, satisfying, and conducive to well-being. Participants emphasized that emotional impact depended partly on their ability to connect with, interpret, or derive meaning from the artwork. Museum staff additionally described examples of exhibitions motivating positive lifestyle changes and personal reflection among visitors. Authors concluded that viewing art within museums may reduce anxiety and enhance subjective well-being through combined effects of aesthetic engagement, reflective experience, calming environmental ambience, and culturally meaningful participation.
Fancourt et al. [62]	Longitudinal cohort study conducted using data from the English Longitudinal Study of Ageing (ELSA) in the United Kingdom. The study involved community-dwelling adults aged ≥50 years followed prospectively over a 10-year period to examine associations between cultural engagement and incident dementia. Participants represented a nationally representative aging population without dementia at baseline and were monitored longitudinally for cognitive outcomes and dementia development.	NeuroArts-related intervention consisted of museum attendance and cultural engagement, specifically participation in museum, gallery, and exhibition visits as forms of passive–interactive visual arts and cultural exposure. Frequency of museum attendance was assessed longitudinally and categorized according to level of cultural engagement. Museum participation involved exposure to visual arts, cultural exhibits, intellectual stimulation, reflective interpretation, social participation, and cognitively enriching environments contributing to cognitive reserve.	Regular museum attendance was associated with a significantly lower incidence of dementia over the 10-year follow-up period. Participants engaging in frequent museum visits demonstrated reduced risk of developing dementia compared with those who never attended museums, even after adjustment for demographic, socioeconomic, health, and social confounders. Findings supported the concept that cultural engagement contributes to cognitive reserve and may exert neuroprotective effects against age-related cognitive decline and dementia. Proposed mechanisms included sustained cognitive stimulation, social interaction, intellectual engagement, emotional enrichment, novelty exposure, reflective processing, and ongoing participation in meaningful cultural activities. Authors concluded that museum attendance may represent a modifiable lifestyle factor supporting healthy cognitive aging and dementia prevention within older adult populations.
Šveb Dragija & Jelinčić [63]	Systematic/narrative review synthesizing 16 records examining psychological well-being outcomes associated with museum engagement across multiple countries and populations. Included studies involved healthy adults, children, older adults, hospital patients, mental health service users, museum visitors, volunteers, and community participants within museums, galleries, heritage institutions, and museum-healthcare partnership settings. The review explored how museum experiences may influence psychological well-being and how museums can be designed to optimize restorative and participatory experiences.	NeuroArts interventions consisted of diverse museum-based visual arts and cultural experiences, including passive art viewing, museum visits, object handling, participatory exhibitions, social prescription museum programmes, horticulture-art museum programmes, interactive multisensory engagement, volunteering, reflective activities, and collaborative museum participation. Interventions emphasized restorative environments, aesthetic engagement, sensory stimulation, cultural participation, object interaction, visitor reflection, social connection, and meaning-making. The review additionally proposed structured museum design principles promoting psychological well-being through attractive, comfortable, comprehensible, participative, innovative, and sustainable museum environments.	Across included studies, museum engagement was associated with enhanced psychological well-being, reduced stress and anxiety, improved mood, increased social connectedness, reflective processing, identity formation, engagement, emotional restoration, and quality of life. Participatory and multisensory museum experiences promoted stronger visitor engagement and emotional impact, while object-handling interventions facilitated reminiscence, identity restoration, and emotional processing. The review identified several environmental and experiential determinants influencing well-being outcomes, including comfort, lighting, sound, participation opportunities, reflective engagement, accessibility, multisensory interaction, and social collaboration. Authors concluded that museums may function as restorative and therapeutic NeuroArts-informed public spaces capable of promoting psychological flourishing and societal wellbeing when intentionally designed to support emotional comfort, participation, reflection, and meaningful cultural engagement.
Wallis & Noble [64]	Qualitative practitioner-led study conducted at the Fitzwilliam Museum involving 10 children aged 3–4 years from a local playgroup participating in a “Playgroup-in-Residence” programme. Children visited the museum repeatedly over the Spring term of 2020 (initially planned for five visits, revised to four due to the COVID-19 pandemic). The study utilized an interpretivist framework, grounded theory methodology, and multimodal data collection, including film recordings, observational notes, surveys, photographs, mark-making artifacts, and spontaneous participant-generated materials.	NeuroArts intervention consisted of repeated museum/gallery interaction and multimodal visual arts engagement within an immersive art museum environment. Children interacted with museum objects, gallery spaces, art materials, display cases, and educators through exploratory play, mark making, drawing, movement, sensory exploration, and participatory dialogue. The intervention emphasized socio-constructivist and post-humanist approaches to meaning-making, recognizing bodily, sensory, material, emotional, and non-verbal interactions as central to children’s engagement with art spaces. Children were encouraged to actively participate in the museum environment, leave marks and traces, interact with physical space, and negotiate their own relationships with the museum through multimodal expression and embodied experience.	Engagement with museum art spaces facilitated children’s sense of ownership, belonging, identity formation, cultural citizenship, and meaning-making. Children used mark-making, movement, sensory exploration, and interactions with objects and spaces to negotiate emotional and social relationships with the museum environment. Findings suggested that museums functioned as multimodal dialogic environments where children actively co-constructed meaning through bodily, sensory, material, and interpersonal interactions rather than solely verbal communication. The repeated museum visits appeared to reduce barriers to participation and fostered confidence, familiarity, and positive relationships between the museum and the local community, particularly among families initially perceiving museums as inaccessible or “too posh.” Authors concluded that participatory visual arts engagement within museum settings may support emotional development, belongingness, identity formation, and community integration among young children through embodied and multisensory interaction with cultural spaces.

**Table 20:** Cultural Interventions in Clinical Populations

Study	Population & Setting	NeuroArts Intervention	Outcomes
Chatterjee et al. [65]	Mixed-methods exploratory study conducted in the United Kingdom involving hospitalized patients, including older adults receiving inpatient care. The project represented a collaboration between healthcare institutions and museum services, examining the therapeutic potential of museum-object engagement in clinical environments. Participants engaged in structured museum-object sessions delivered within hospital settings.	NeuroArts intervention consisted of tactile museum-object handling sessions using authentic museum artifacts and heritage objects. Participants were invited to physically handle, observe, discuss, and reflect upon museum objects facilitated by museum staff and programme facilitators. Sessions emphasized multisensory stimulation, tactile exploration, curiosity, reminiscence, storytelling, emotional engagement, and social interaction. The intervention intentionally moved beyond passive art observation toward active embodied interaction with culturally meaningful objects within healthcare settings.	Museum-object handling was associated with improvements in life satisfaction, subjective health perception, emotional well-being, reminiscence, social engagement, and identity restoration among hospitalized participants. Participants reported heightened curiosity, enjoyment, emotional stimulation, reflective thinking, and opportunities for meaningful interpersonal interaction and autobiographical storytelling. The tactile and multisensory nature of the intervention appeared to facilitate distraction from illness, cognitive engagement, memory retrieval, and emotional processing. Findings suggested that active engagement with museum artifacts may provide therapeutic benefits through mechanisms involving sensory stimulation, reflective meaning-making, narrative recall, interpersonal dialogue, and restoration of personal identity within clinical settings.
Zeilig et al. [66]	Systematic review synthesizing studies involving individuals with mild-to-moderate dementia participating in museum- and gallery-based programmes across community and cultural settings. Included studies evaluated older adults with dementia engaged in structured museum interventions delivered through collaborations between museums, galleries, healthcare services, and community organizations. Programmes frequently incorporated caregivers, museum educators, facilitators, artists, and group-based participation.	NeuroArts interventions consisted of museum-based visual arts programmes, including guided museum/gallery tours, facilitated art discussions, object handling, reminiscence activities, participatory visual arts engagement, multisensory cultural experiences, and socially interactive museum sessions. Interventions emphasized aesthetic engagement, emotional reflection, autobiographical memory stimulation, cognitive activation, sensory enrichment, interpersonal dialogue, and socially meaningful participation within culturally rich environments.	Museum-based programmes were associated with improvements in mood, psychological wellbeing, cognition, communication, confidence, social interaction, engagement, and quality of life among individuals with mild-to-moderate dementia. Participants demonstrated enhanced attention, emotional expression, reminiscence, interpersonal connection, and social participation during and after programme engagement. Object handling and facilitated discussions of artwork particularly promoted autobiographical recall, identity reinforcement, and meaningful communication. Caregivers additionally reported improved relational interaction, shared enjoyment, and reduced social isolation. Findings supported museum-based NeuroArts interventions as promising non-pharmacologic, person-centered approaches for dementia care through mechanisms involving cognitive stimulation, sensory engagement, reflective processing, emotional enrichment, and socially meaningful artistic participation.
Beauchet et al. [67]	An experimental study conducted in Montreal, Canada, involving adult participants recruited through a physician-prescribed museum visit programme developed through collaboration between healthcare providers and the Montreal Museum of Fine Arts. Participants were adults receiving social prescriptions for museum attendance as part of an innovative non-pharmacologic wellbeing intervention integrated into healthcare delivery. The study evaluated mental health and quality-of-life outcomes following structured museum exposure.	NeuroArts intervention consisted of physician-prescribed museum visits, wherein participants attended museum exhibitions and engaged in passive–interactive visual arts experiences within a cultural environment. Participants explored galleries, viewed artworks, and immersed themselves in museum spaces, emphasizing aesthetic appreciation, reflective engagement, emotional processing, intellectual stimulation, and cultural participation. The intervention represented a social-prescribing model linking healthcare systems with arts and cultural institutions to promote mental well-being through structured exposure to visual arts environments.	Museum visits resulted in significant improvements in subjective well-being and quality-of-life measures among participants. Participants reported enhanced emotional well-being, relaxation, positive mood, psychological restoration, and meaningful engagement following museum attendance. Findings supported the feasibility and therapeutic value of museum prescriptions as adjunctive psychosocial interventions integrated into healthcare pathways. The authors proposed that museum exposure may exert beneficial effects through mechanisms involving stress reduction, cognitive stimulation, emotional enrichment, reflective meaning-making, social participation, and restorative engagement with artistic environments. The study concluded that physician-prescribed museum visits may represent an effective NeuroArts-informed social-prescribing strategy to support mental health and wellbeing within community healthcare frameworks.

**Table 21:** Online, Digital, and Electronic Arts in Healthy Populations

Study	Population & Setting	NeuroArts Intervention	Outcomes
Trupp et al. [68]	An experimental online study conducted in Austria involving adult participants recruited from the general population during the COVID-19 pandemic. Participants engaged remotely with online art and cultural presentations delivered through digital platforms. The study examined whether brief exposure to online digital art experiences could influence psychological well-being outcomes in healthy adults under socially restrictive conditions.	NeuroArts intervention consisted of online digital visual arts exposure through brief interactions with digital art and culture presentations. Participants viewed curated online artistic and cultural content, including museum-based visual art presentations and digital cultural experiences delivered remotely. Interventions emphasized aesthetic engagement, reflective observation, emotional immersion, cultural participation, and cognitive stimulation through online visual arts platforms. Comparator conditions included non-art online content exposure.	Brief exposure to online digital art significantly improved mood, reduced state anxiety and loneliness, and enhanced subjective well-being among participants. Participants demonstrated immediate positive emotional changes following engagement with online artistic and cultural content. Findings suggested that even short-duration digital visual arts exposure may exert beneficial psychological effects through mechanisms involving emotional engagement, aesthetic appreciation, distraction, reflective processing, and perceived social-cultural connectedness. The study supported the feasibility and therapeutic potential of accessible online NeuroArts interventions, particularly during periods of social isolation and reduced in-person cultural engagement.

**Table 22:** Online, Digital, and Electronic Arts in Clinical Populations

Study	Population & Setting	NeuroArts Intervention	Outcomes
McCabe et al. [69]	Randomized controlled trial conducted in Ireland involving adult patients undergoing hematopoietic stem cell transplantation within hospital inpatient transplant units. Participants experienced prolonged hospitalization, protective isolation, intensive treatment-related stress, anxiety, and reduced quality of life associated with stem cell transplantation. The study evaluated the therapeutic effects of immersive digital visual art integrated into clinical care environments.	NeuroArts intervention consisted of digital visual arts / new media art delivered through a “virtual window” installation within patient hospital rooms. The intervention utilized large-screen dynamic digital audiovisual displays presenting continuously changing nature scenes and artistic environmental imagery designed to simulate exposure to outdoor environments and aesthetically restorative spaces. The intervention emphasized sensory enrichment, emotional restoration, environmental enhancement, aesthetic engagement, distraction, relaxation, and reduction of environmental monotony during prolonged hospitalization and isolation.	The digital art intervention was associated with reductions in anxiety and depression and improvements in quality of life and overall patient experience among stem cell transplantation patients. Participants reported enhanced emotional well-being, improved environmental comfort, reduced psychological distress, and more positive perceptions of the hospital environment. The intervention appeared to mitigate feelings of confinement, sensory deprivation, and emotional burden associated with prolonged inpatient isolation. Findings supported the feasibility and therapeutic potential of immersive digital visual arts interventions within highly medicalized clinical settings. Proposed mechanisms included distraction, sensory stimulation, emotional regulation, environmental enhancement, restorative visual exposure, and simulated connection with aesthetically meaningful natural environments.
Sangeorzan et al. [70]	Qualitative interpretative phenomenological analysis conducted in the United Kingdom involving individuals with severe mental illness who created and uploaded personal vlogs on YouTube. Participants were adults publicly sharing lived experiences of psychiatric illness through digital video blogging platforms. Diagnoses included severe mental health conditions such as psychotic disorders, bipolar disorder, major depression, and related psychiatric illnesses. The study explored the subjective experiences and perceived psychosocial effects of mental health vlogging.	NeuroArts intervention consisted of digital narrative arts through video blogging (vlogging) on YouTube. Participants created autobiographical digital narratives discussing personal experiences of severe mental illness, recovery, coping, stigma, treatment, identity, and daily life. Vlogging involved multimodal artistic self-expression integrating spoken narrative, visual presentation, audiovisual storytelling, performance, editing, reflective communication, and online social interaction. The digital platform additionally enabled peer communication, audience engagement, reciprocal support, and public sharing of lived experiences.	Vlogging facilitated peer support, reduced social isolation and stigma, enhanced self-efficacy, promoted emotional expression, and supported recovery-oriented identity formation among participants. Participants described vlogging as providing opportunities for validation, connection, empowerment, self-reflection, advocacy, and communication of experiences that were otherwise difficult to express. The online platform enabled the formation of supportive virtual communities and fostered feelings of belongingness and mutual understanding among individuals with severe mental illness. Participants additionally reported therapeutic benefits related to narrative meaning-making, emotional processing, confidence-building, and reclaiming agency over personal illness narratives. Findings suggested that digital narrative arts through vlogging may represent a novel NeuroArts modality supporting psychosocial recovery, self-expression, and community-building within severe mental illness populations.
